# Dynamic Optimization and Coordination of Cooperative Emission Reduction in a Dual-Channel Supply Chain Considering Reference Low-Carbon Effect and Low-Carbon Goodwill

**DOI:** 10.3390/ijerph18020539

**Published:** 2021-01-11

**Authors:** Ziyuan Zhang, Liying Yu

**Affiliations:** School of Management, Shanghai University, Shanghai 200444, China; zhangziyuan@shu.edu.cn

**Keywords:** reference low-carbon effect, low-carbon goodwill, dual-channel supply chain, cooperative emission reduction, coordination contract, differential game

## Abstract

Although the issue of cooperative emission reduction in supply chains has been extensively studied, there is little literature that considers the impact of consumers’ reference low-carbon effect and product low-carbon goodwill on their purchasing behavior in the issue of dual-channel supply chain cooperative emission reduction. In order to explore the impact of consumers’ reference low-carbon effect and product low-carbon goodwill on the balanced emission reduction decisions and profit of dual-channel supply chain members, we establish a dual-channel supply chain emission reduction dynamic optimization model, use differential game theory to solve the manufacturer’s optimal emission reduction investment and the retailer’s optimal low-carbon publicity investment strategies under four different decision scenarios, and analyze them in detail. In addition, we also design an effective low-carbon publicity cost-sharing contract to achieve coordination of the supply chain. The research results show that the equilibrium strategies of the manufacturer and retailer and the overall profit of the supply chain under the centralized decision scenario are better than those of decentralized decision scenario. When the initial reference low-carbon level is low, the online and offline reference low-carbon effects are beneficial to the manufacturer and retailer. When the initial low-carbon goodwill is high, it is beneficial for both the manufacturer and retailer to increase consumer recognition of low-carbon goodwill. When the ratio of low-carbon publicity cost sharing provided by the manufacturer to the retailer is within a reasonable range, the cost-sharing contract can reduce the double marginal effect and achieve supply chain coordination.

## 1. Introduction

The fifth assessment report of the United Nations Intergovernmental Panel on Climate Change (IPCC) pointed out that in the past 150 years, the global average temperature has risen by about 0.8 °C, which has caused a series of droughts, floods, famines, and environmental and social issues. Global warming has brought severe challenges to the survival and development of human. That the emission of greenhouse gases (mainly carbon dioxide) is the main cause of global warming has been universally recognized by the international community. Under the pressure of tremendous changes in the global climate, a low-carbon economic development model based on the concept of low pollution, low energy consumption, and sustainable development has become a hot spot of international society. Subsequently, in order to promote the low-carbon economic development model around the world, various joint conventions and environmental protection regulations have been issued one after another. The Copenhagen Climate Change Conference held in December 2009 discussed the 2012–2020 global emission reduction agreement, which clearly requires all countries in the world to reach certain carbon emission reduction targets, and enterprises in various countries will, therefore, face tremendous pressure to reduce emissions. At the same time, the deterioration of the ecological environment has further aroused the public’s awareness of environmental protection. Energy-saving and emission-reduction products are increasingly being respected by consumers [1]. For example, a survey of consumers in America showed that about 80% of consumers would buy low-carbon products even if they need to pay an additional fee [2]. Facing the fiercely competitive market environment, actively reducing emissions will be an important way for companies to gain competitive advantages. Currently, in order to gain stronger brand competitiveness and greater market share, more and more companies are racing to develop low-carbon products such as fluorine-free inverter air conditioners and refrigerators with first-class energy efficiency standards, such as Galanz, Haier, Midea, and other home appliance companies. Therefore, it is of great practical significance to discuss how companies can effectively reduce emissions in the context of low-carbon economy.

As the online retail market matures, e-commerce and other Internet economies are booming; online shopping and consumption have become the first choice of contemporary consumers, especially young consumers. For example, according to the 45th China Statistical Report on Internet Development, the national online retail sales reached CNY 10.63 trillion in 2019, which is equivalent to the total national GDP in 2001! The number of online shopping users in China reached 710 million, an increase of 16.4% from the end of 2018, accounting for 78.6% of the total netizens, and the per capita annual consumption was more than CNY 10,000 [3]! Due to the impact of the online retail market, traditional manufacturing companies have to accelerate the pace of strategic transformation, build e-commerce channels, and form an online and offline dual-channel business model [4]. For example, traditional manufacturing companies represented by China’s home appliance giant Gree Electric have improved offline traditional retail channels in recent years, while also starting the construction of e-commerce channels, focusing on creating online direct sales channels. Gree Electric’s annual report shows that after the opening of online direct sales channels, during the Double Eleven period in 2018 alone, Gree air conditioners achieved sales of CNY 1.259 billion across the network, accounting for 28.1% of the market. In 2019, the online channels of Gree Electric Appliances were even stronger during the “Double Eleven”. On that day, the sales of all categories on the whole network exceeded CNY 4.1 billion, a year-on-year increase of over 200% [5]. The above example show that the manufacturer’s opening of online direct sales channels has brought huge returns to them, and a large number of studies have confirmed that manufacturers can make the supply chain perform better by establishing online direct sales channels [6].

Therefore, as the low-carbon economy and the dual-channel sales model of e-commerce have become the general trend, in order to cater to consumers’ increasingly strong low-carbon consumption concepts and online consumption concepts, further expand product market demand, manufacturing companies in the supply chain have also begun to join forces with retail companies to reduce emissions while actively opening up their own network sales channels. On the one hand, manufacturing companies have adopted measures such as strengthening the research of low-carbon technology to produce low-carbon products with a higher low-carbon degree. For example, well-known sports brand manufacturer Adidas conducts low-carbon processing on product manufacturing materials and packaging materials. Best Buy, the world’s largest home appliance retailer, saves energy and protects the environment by selling Energy Star, a product with superior energy efficiency [7]. On the other hand, retail companies use offline low-carbon promotion to attract consumers to purchase low-carbon products. For example, China’s Suning and Gome highlights low-carbon products through store display and a large number of posters. Walmart sets up special areas in shopping malls and cooperates with suppliers to provide low-carbon lifestyle education promotion activities [8]. The above examples and numerous studies have confirmed that cooperation between manufacturers and retailers in emission reduction activities has become an effective way of cooperation in the context of a low-carbon economy. Therefore, it is of great practical significance to study the members’ cooperative emission reduction issues of a dual-channel supply chain in the context of low-carbon economy.

Low-carbon consumption is an important link in realizing the development of a low-carbon economy. Consumers’ low-carbon consumption concepts are playing an increasingly important role on the road of energy conservation and emission reduction in enterprises. In order to obtain stronger market competitiveness and greater market share, companies must thoroughly study consumers’ behavior characteristics and seriously consider them when formulating operational strategies. At present, scholars have conducted a series of studies on consumers’ purchasing behaviors and found that consumers’ purchasing behaviors are not only affected by the current attributes of the products, but also related to the expectations of the attributes of the products formed by their previous purchase experiences. Scholars define this psychology as the reference effect [9,10]. Undoubtedly, consumers with low-carbon preference are not only affected by the current value of emission reduction efforts when they choose to purchase low-carbon products, but also affected by the reference point of emission reduction efforts formed from the information collected in the past purchase experience. They will compare the company’s emission reduction efforts with the reference point. When it is higher than the reference point, a sense of gain will be formed, otherwise a sense of loss will be formed, that is, reference low-carbon effect. Because the reference low-carbon effect will affect consumers’ purchase behavior of low-carbon products, it is necessary to introduce it into the study of joint emission reduction issue in the supply chain to explore its impact on enterprises’ emission reduction and low-carbon publicity decisions.

Based on the above background, this paper studies the joint emission reduction issue of manufacturers and retailers in a manufacturer-led dual-channel supply chain considering consumers’ reference low-carbon effect and product low-carbon goodwill by using the Stackelberg differential game method. We are mainly committed to solving the following problems:

Considering the differential influence of consumers’ reference low-carbon effect and product low-carbon goodwill on the product demand of online and offline channels, what are the optimal emission reduction investment decisions of the manufacturer and the retailer under different decision scenarios? What is the difference between the optimal decisions under centralized and different decentralized decision scenarios? 

How do consumers’ reference low-carbon effect and low-carbon goodwill jointly affect both two parties’ optimal emission reduction investment strategies, profits, and the total profit of supply chain?

What is the optimal path for low-carbon goodwill and reference low-carbon level over time under different initial low-carbon goodwill and reference low-carbon levels? Under different initial reference low-carbon levels and low-carbon goodwill, what impact will consumers’ reference low-carbon effect and low-carbon goodwill have on the profits of both parties?

Compared with not sharing the emission reduction costs with each other, what impact will the sharing of emission reduction costs between the two parties have on the emission reduction investment of both parties? How can we design an effective contract to achieve supply chain coordination under such circumstances?

The main contributions of this paper are as follows.

From a theoretical point of view, since few studies have introduced consumers’ reference low-carbon effect into the issue of joint emission reduction in a dual-channel supply chain, considering the differential influence of consumers’ reference low-carbon effect and product low-carbon goodwill on the product demand of online and offline channels and integrating reference low-carbon effect and low-carbon goodwill to discuss the joint emission reduction decisions of manufacturers and retailers, this paper investigates in-depth the emission reduction strategies of a dual-channel supply chain in consideration of consumers’ reference low-carbon effect and low-carbon goodwill. Therefore, this paper enriches the research content of the joint emission reduction problem of the dual-channel supply chain. From a practical point of view, as the low-carbon economy and e-commerce economy are increasingly prosperous, facing the pressure of emission reduction, more and more traditional manufacturers have established online direct sales channels and cooperated with retailers to carry out emission reduction activities. In this context, this paper has launched research on the joint emission reduction issue in a dual-channel supply chain considering the impact of consumer reference low-carbon effects and low-carbon goodwill, and the conclusions of the research can provide manufacturers, retailers, and supply chain consultants with some useful suggestions to make suitable emission reduction decisions. 

The remainder of the paper is arranged as follows: Section 2 is the literature review. Problem description and research assumptions are provided in Section 3. Section 4 presents optimal decisions under four decision scenarios. Section 5 entails analysis and discussion. It draws on some research conclusions by comparing the equilibrium solutions obtained from Section 4. Section 6 provides a supply chain coordination mechanism. Some numerical computations are given in Section 7 to test and verify our findings. The conclusion and limitations of the study are summed up in Section 8.

## 2. Literature Review 

In this section, we mainly review two types of literature about supply chain operation decision issues according to the research content. One stream is about joint emission reduction issues of supply chain, another one is about supply chain operation decision issues considering reference effect.

In the context of a low-carbon economy, the issue of joint emission reduction in the supply chain has received extensive attention from scholars around the world [11]. Wang et al. [12] studied the issue of emission reduction and coordination in the low-carbon supply chain led by retailers based on consumers’ low-carbon preference. Considering the fairness concerns of retailers and the low-carbon preferences of consumers, Zhou et al. [8] further investigated the coordination of the low-carbon supply chain based on advertising and abatement cost-sharing contracts. Pu et al. [13] discussed the competition among supply chains and governmental policy considering consumers’ low-carbon preference. Peng et al. [14] further studied the coordination contracts for a supply chain considering yield uncertainty and low-carbon preference. Li et al. [15] investigated price and carbon emission reduction decisions considering fairness concerns and designed a revenue-sharing contract. Considering strategic customer behavior and green technology investment, Jiang et al. [16] discussed optimal strategies for low carbon supply chain. Han et al. [17] studied the optimal decisions in a low-carbon e-supply chain considering the manufacturer’s carbon emission reduction behavior. Considering carbon concerned demand, Du et al. [18] investigated performance and policies of low-carbon supply. Zhang et al. [19] discussed carbon emission reduction decisions of low-carbon supply chain members with retailer’s fairness concern and subsidies. Under cap-and-trade regulation policy, Wang et al. [20] studied production and joint emission reduction decisions and designed a two-way cost-sharing contract. Based on the carbon footprint of the product, Wang et al. [21] discussed low-carbon supply chain members’ joint emission reduction decisions. Considering consumers’ low-carbon preference, Zhang et al. [22] investigated joint emission reduction decisions of a dual-channel low-carbon supply chain and designed a BOPS (Designing Buy-Online-and-Pick-Up-in-Store) contract to coordinate the dual-channel low-carbon supply chain. Considering the lag time of emission reduction technologies and low-carbon preference of consumers, Sun et al. [23] studied carbon emission transfer strategies in supply chain. Based on financial constraints, Cao et al. [24] discussed the optimal carbon reduction decisions and ordering quantity of low-carbon supply chain members. Considering government carbon subsidies and fairness concerns, Han et al. [25] investigated the optimal decisions and coordination of low-carbon e-commerce supply chain. Considering the competition between manufacturing and remanufacturing products, Rezaei et al. studied the sustainable decisions in a closed-loop supply chain with a competitive environment [26].

Analyzing and summarizing the above literature, we can see some literature studied consumers’ low-carbon preference [12,14,15,23], some studied government regulations [19,20,25], some studied supply chain members’ behavior preference [15,19], some discussed dual-channel and supply chain competition [13,22,26], and some investigated supply chain coordination [12,14,20,25]. However, the low-carbon supply chain optimization models that the above literature establish are all static low-carbon supply chain optimization models. The equilibrium results obtained from the static decision models can only reflect the short-term optimal decisions of enterprises, while the dynamic decision models can better reflect the long-term effects of enterprises’ emission reductions. 

There is also some literature that studied emission reduction issues by using the dynamic optimization models. Considering social preferences of supply chain members, Xia et al. [27] studied joint emission reduction issues by using differential game theory. Guan et al. [28] studied a supply chain coordination problem with a manufacturer and a retailer that have Nash bargaining fairness concerns. Yu et al. [29] established the optimal control model, researched the joint emission reduction issue, and designed a new coordination contract. 

Although the above studies used dynamic optimization models to study joint emission reduction issues, the above literature does not consider consumers’ reference low-carbon effect, so the research content needs to be supplemented.

In recent years, there have been many studies about supply chain operation issues with the consideration of reference effect. In terms of reference price effect, Popescu et al. [30] discussed the dynamic pricing problem under the reference price effect. In the coordination of supply chain, Zhang et al. [31] considered the advertising and consumers’ reference price effect and used the bilateral cost-sharing contract to achieve the coordination of the supply chain. Considering reference price effect, Saha et al. [32] discussed the optimal investment decisions of green operation and protection technology of perishable products. Considering reference price effect, Dye et al. [9] studied the optimal investment problem of joint dynamic pricing and technology protection of integrated supply chain. Lin [33] discussed price promotion problem in supply chain with the reference price effect. Considering reference price effect, Xu et al. [34] studied the optimal decisions of closed loop supply chain. Zhang et al. [35] took the reference price effect into the competitive supply chain and discussed supply chain members’ strategic pricing decisions. In terms of reference quality effect, He et al. [36] investigated the coordination of the supply chain. In a revenue-sharing supply chain, Liu et al. [10] studied myopic and far-sighted behaviors of supply chain members with the consideration of reference quality effect. Based on a closed-loop supply chain, Zhang et al. [37] discussed static and dynamic pricing strategies of supply chain members considering reference quality effect. Considering both reference product quality effect and reference service quality effect, Zhou et al. [38] investigated the quality decisions and coordination of a dual-channel supply chain. Based on the O2O environment, He et al. [39] studied the optimal decisions of the supply chain with reference quality effect.

It can be seen from the above literature that the current research on the reference effect mostly focuses on the reference price effect [30,31,32,33,34,35], and a few studies involve the reference quality effect [10,36,37,38,39], but few studies have involved consumers’ reference low-carbon effect. As the low-carbon economy and e-commerce economy mature, consumers’ low-carbon consumption concepts and online consumption concepts have become increasingly prominent, and consumers’ reference low-carbon effect will become more prominent and will have a significant impact on consumers’ buying behavior. In addition, as more and more traditional manufacturers establish online direct sales channels and actively cooperate with retailers to reduce carbon emissions, it is very necessary to discuss the issue of joint emission reductions between manufacturers and retailers in the context of dual channels and design effective contracts to achieve supply chain coordination.

Considering the shortcomings of previous studies and based on the actual situation, we think there still exists some work to be done. 

Therefore, based on a dual-channel supply chain led by the manufacturer, comprehensively considering the differential impact of consumers’ reference low-carbon effect and product low-carbon goodwill on the product demand of online and offline channel, we will use differential game theory to explore how consumers’ reference low-carbon effect and low-carbon goodwill jointly influence the manufacturer’s optimal emission reduction investment, the retailer’s optimal low-carbon publicity investment, and both parties’ profit and supply chain total profit under different scenarios. Moreover, we will also conduct detailed comparison and analysis of relevant equilibrium results and design an effective contract to coordinate the dual-channel supply chain.

So as to further highlight the innovation of our paper and clearly reveal the difference between previous literature and our paper, we will enumerate some relevant literature in Table 1. We mainly use the reference low-carbon effect, dual channel, low-carbon goodwill, and the coordination contract as the classification criteria according to our research content to achieve the purpose of differentiation. Besides, it should be noted that Table 1 is just a rough division about this literature, there still exist some differences between them and our paper due to different research priorities.

Based on the information of Table 1, it can be summarized that previous literature relevant to this paper only considered one of the following factors, such as reference low-carbon effect, low-carbon goodwill, dual channel, and coordination mechanism. However, the above factors are the basis of this paper; therefore, this paper combines them together and studies the impact of consumers’ reference low-carbon effect and product low-carbon goodwill on the emission reduction strategies of manufacturers and retailers in a dual-channel supply chain. 

## 3. Problem Description and Research Assumptions

Consider a two-tier supply chain consisting of only one manufacturer and one retailer. The manufacturer invests in emission reduction to produce a certain low-carbon product, and the higher the manufacturer’s investment in emission reduction, the higher the low-carbon level and environmentally friendliness of the product. At the same time, in order to attract consumers to buy this low-carbon product, the retailer will also conduct low-carbon promotion in a variety of ways. The manufacturer can sell low-carbon products through the offline retailer and its online direct sales channel, and there is no competition between the two channels to simplify the model. See Table 2 for the information of decision variables and major parameters.

To facilitate subsequent study, we put forward some assumptions presented as follows: 

(1) Since most manufacturers still occupy a dominant position in the market, we assume that manufacturers occupy a dominant position in the supply chain system. Manufacturers and retailers are both rational decision makers, both have complete market information and both parties play a master–slave game of Stackelberg with the goal of maximizing their own interests. 

(2) It is assumed that consumers’ past consumption experiences will promote consumers to form low-carbon expectations for the low-carbon products and cause a significant impact on their next low-carbon consumption, that is, reference low-carbon effect. According to Dye et al. [9] and Liu et al. [10], we can define the reference low-carbon effect as the reference point of the product low-carbon level formed by consumers in the long-term purchase activity. Learn from previous literature [45], the consumer’s reference low-carbon level R(t) is defined as the weighted average of the product’s past low-carbon levels, that is, $R\left(t\right)=\varepsilon \int_{0}^{t}e^{\varepsilon \left(s-t\right)}E\left(s\right)ds$. Taking the derivative of this formula and applying Leibniz’s law, we can get the following differential equation shown in Equation (1). Therefore, we use the differential equation shown in Equation (1) to describe the dynamic change process of consumers’ reference low-carbon levels.

(1) $$\begin{array}{l} \dot{R}\left(t\right)=\varepsilon \left(E\left(t\right)-R\left(t\right)\right)\\ R\left(0\right)=R_{0} \end{array}$$

where R(t) represent the reference low-carbon level at time t, and the initial reference low-carbon level is $R\left(0\right)=R_{0}$, E(t) is the emission reduction investment at time t determined by the manufacturer, ε>0 is the memory parameter, when ε is larger, it means that consumers are biased towards short-term memory of the low-carbon level of the product and have low loyalty.

(3) It is assumed that the low-carbon promotion of retailers can not only attract consumers to buy in the short term, but also help the product establish a brand image, that is, low-carbon goodwill, in the long run. Based on the previous literature [46,47] and referring to Nerlove–Arrow’s classic goodwill model, we use the differential equation shown in Equation (2) to describe the dynamic change process of product low-carbon goodwill.

(2) $$\begin{array}{l} \dot{G}\left(t\right)=\lambda A\left(t\right)-\sigma G\left(t\right)\\ G\left(0\right)=G_{0} \end{array}$$

where G(t) represent the low-carbon goodwill level at time t, and the initial low-carbon goodwill level is $G\left(0\right)=G_{0}$, A(t) is the low-carbon publicity investment at time t determined by the retailer, λ>0 indicates the influence coefficient of the retailer’s low-carbon publicity on the low-carbon goodwill, σ>0 indicates the natural decay rate of low-carbon goodwill, which is usually due to the impact of new product launches and consumer forgetting.

(4) Based on the previous literature [48], it is assumed that the demand function of low-carbon products is a linear function. Consumers tend to buy products with higher low-carbon degree, low-carbon publicity, and low-carbon goodwill, and they can choose any channel to buy products. Due to the reference low-carbon effect, when consumers buy products, they will compare the actual low-carbon level of the product with the reference low-carbon level. If the actual low-carbon level at this time is higher than the reference low-carbon level, a sense of gain will be formed in their mind and they will be willing to buy the product, otherwise a sense of loss will be formed. In addition, it is assumed that retailers’ offline low-carbon promotion will have a positive spillover effect on online demand. What needs special explanation is that since this paper is a long-term study, it does not consider the impact of price changes. Based on the above assumptions, the demand for products in each channel is mainly affected by consumers’ reference low-carbon effect, product low-carbon goodwill, manufacturer’s emission reduction level, and retailer’s low-carbon publicity investment; therefore, the demand functions of online channel and offline channel are as follows:

(3) $$Q_{d}=d_{d}+\alpha_{d}\left[E\left(t\right)-R\left(t\right)\right]+\beta_{d}E\left(t\right)+\mu G\left(t\right)+\xi kA\left(t\right)$$

(4) $$Q_{r}=d_{r}+\beta_{r}E\left(t\right)+kA\left(t\right)+\alpha_{r}\left[E\left(t\right)-R\left(t\right)\right]+\mu G\left(t\right)$$

where $Q_{d}$ is the product demand of online channel, $Q_{r}$ is the product demand of the offline channel. $d_{d}$, $d_{r}>0$, respectively, indicate the potential market demand in online channel and offline channels. $\alpha_{d}$, $\alpha_{r}>0$, respectively, indicate the influence coefficient of consumers’ reference low-carbon effect on the demand of online and offline channels. $\beta_{d}$, $\beta_{r}>0$, respectively, indicate the influence coefficient of manufacturer’s emission reduction level on the demand of online and offline channels. μ>0 indicate the influence coefficient of product low-carbon goodwill on the demand of online and offline channels. k>0 indicates the influence coefficient of retailer’s offline low-carbon promotion on the product demand of the offline channel. 0≤ξ≤1 indicates the indirect spillover effect coefficient of retailer’s offline low-carbon promotion on the demand of the online direct sales channel: the larger the value of ξ, the better the spillover effect.

(5) Similar to the previous literature [49], let the manufacturer’s emission reduction cost and retailer’s low-carbon promotion cost be convex functions of emission reduction investment and low-carbon promotion investment, respectively. That is, the manufacturer’s emission reduction cost is $C_{m}=\frac{1}{2}\eta_{m}E^{2}\left(t\right)$, where $\eta_{m}>0$ represents emission reduction cost coefficient; the retailer’s low-carbon promotion cost is $C_{r}=\frac{1}{2}\eta_{r}A^{2}\left(t\right)$, where $\eta_{r}>0$ represents low-carbon promotion cost coefficient.

(6) Since the impact of product prices is not considered, we assume that the manufacturer’s marginal profits for online and offline channels are $\pi_{md}$, $\pi_{mr}$, respectively, the retailer’s marginal profit is $\pi_{rr}$, $\pi_{md}$, $\pi_{mr}$, $\pi_{rr}$ are assumed to be exogenous variables. Within a business scope of unlimited time, the manufacturer and retailer have the same discount rate ρ (ρ>0) at any time. Both parties make rational decisions based on complete information, and their decision goals are to maximize their own profits. In addition, we do not consider the cost of inventory in the supply chain to simplify the model.

The decision variables and major parameters in our research are presented in Table 2.

## 4. Model Construction and Solution

Based on the research hypothesis and basic model in the previous section, this section will mainly consider four decision scenarios: decentralized decision scenario without cost-sharing(DN), decentralized decision scenario with cost sharing (DD), decentralized decision scenario with bilateral cost sharing (DC), and centralized decision scenario (CC). Under each decision scenario, we will solve the manufacturer’s optimal emission reduction investment strategy, the retailer’s optimal low-carbon promotion investment strategy, the optimal profit of both parties, the optimal dynamic evolution trajectory of product low-carbon goodwill, and consumers’ reference low-carbon level. To facilitate the distinction, we will use superscripts n, d, c, cc to denote the four decision scenarios: decentralized decision scenario without cost sharing (DN), decentralized decision scenario with cost-sharing (DD), decentralized decision scenario with bilateral cost sharing (DC), and centralized decision scenario (CC). Besides, we will also use the subscripts m, r, sc to, respectively, represent the decision maker: manufacturer, retailer, and the whole supply chain. At the same time, the time will not be listed below for the convenience of variable writing. 

Next, we will use the Hamilton–Jacobi–Bellman equation (hereinafter referred to as the HJB equation) to solve the equilibrium results of the models, and learning from Jørgensena [50], we assume that the parameters in the model are constants that have nothing to do with time.

### 4.1. Decentralized Decision Scenario Without Cost-Sharing (DN)

Under this decision scenario, the manufacturer first determines its own emission reduction investment strategy, sells products to the retailer with marginal profit $\pi_{mr}$, and meanwhile, sells products directly to consumers through online direct sales channel with marginal profit $\pi_{md}$. After determining its own low-carbon promotion strategy, the retailer sells the products purchased from the manufacturer to consumers with marginal profit $\pi_{rr}$. At this time, the objective functions of the manufacturer and retailer are as follows:

(5) $$J_{m}^{n}\left(G,R,t\right)=\underset{E}{\max}\int_{0}^{\infty }e^{-\rho t}\left[\pi_{mr}\left[\begin{array}{l} d_{r}+\left(\alpha_{r}+\beta_{r}\right)E\\ +kA+\mu G-\alpha_{r}R \end{array}\right]+\pi_{md}\left[\begin{array}{l} d_{d}+\left(\alpha_{d}+\beta_{d}\right)E\\ +\mu G+\xi kA-\alpha_{d}R \end{array}\right]-\frac{\eta_{m}E^{2}}{2}\right]dt$$

(6) $$J_{r}^{n}\left(G,R,t\right)=\underset{A}{\max}\int_{0}^{\infty }e^{-\rho t}\left[\pi_{rr}\left[d_{r}+\left(\alpha_{r}+\beta_{r}\right)E+kA+\mu G-\alpha_{r}R\right]-\frac{\eta_{r}A^{2}}{2}\right]dt$$

**Theorem** **1.** *Under decentralized decision scenario without cost-sharing (DN), we can get the following results by solving the decision model:*

*(1)*  *Differential game equilibrium strategies of the manufacturer and retailer are as follows:*
  $$\left\{ \begin{array}{l} E^{n*}=\frac{\pi_{mr}\left[\rho \left(\alpha_{r}+\beta_{r}\right)+\varepsilon \beta_{r}\right]+\pi_{md}\left[\rho \left(\alpha_{d}+\beta_{d}\right)+\varepsilon \beta_{d}\right]}{\eta_{m}\left(\rho +\varepsilon \right)}\\ A^{n*}=\frac{\pi_{rr}\left[k\left(\rho +\sigma \right)+\lambda \mu \right]}{\eta_{r}\left(\rho +\sigma \right)} \end{array}\right.$$
*(2)*  *The optimal evolution trajectory of low-carbon goodwill is as follows:*
  $$G^{n}\left(t\right)=\left(G_{0}-C^{n}\right)e^{-\sigma t}+C^{n},C^{n}=\frac{\lambda A^{n*}}{\sigma }$$
*(3)*  *The optimal evolution trajectory of the reference low-carbon level is as follows:*
  $$R^{n}\left(t\right)=\left(R_{0}-E^{n*}\right)e^{-\varepsilon t}+E^{n*}$$
*(4)*  *The present value functions of manufacturer’s optimal profit and retailer’s optimal profit are as follows:*
  $$\begin{array}{l} J_{m}^{n*}=\frac{1}{\rho }\pi_{mr}\left(d_{r}+\beta_{r}E^{n*}+kA^{n*}\right)+\frac{1}{\sigma \left(\rho +\sigma \right)}\pi_{mr}\mu \left(G_{0}-\lambda A^{n*}\right)-\frac{1}{\left(\rho +\varepsilon \right)}\pi_{mr}\alpha_{r}\left(R_{0}-E^{n*}\right)\\ +\frac{1}{\rho }\pi_{md}\left(d_{d}+\beta_{d}E^{n*}+\xi kA^{n*}\right)+\frac{1}{\sigma \left(\rho +\sigma \right)}\pi_{md}\mu \left(G_{0}-\lambda A^{n*}\right)-\frac{1}{\left(\rho +\varepsilon \right)}\pi_{md}\alpha_{d}\left(R_{0}-E^{n*}\right)-\frac{\eta_{m}E^{n*}{}^{2}}{2\rho } \end{array}\;J_{r}^{n*}=\frac{1}{\rho }\pi_{rr}\left(d_{r}+\beta_{r}E^{n*}+kA^{n*}\right)+\frac{1}{\sigma \left(\rho +\sigma \right)}\pi_{rr}\mu \left(G_{0}-\lambda A^{n*}\right)-\frac{1}{\left(\rho +\varepsilon \right)}\pi_{rr}\alpha_{r}\left(R_{0}-E^{n*}\right)-\frac{\eta_{r}A^{n*}{}^{2}}{2\rho }$$
  *Proof (See Appendix A).*

Theorem 1 shows that under the decentralized decision scenario without cost-sharing (DN), manufacturers and retailers often make decisions based on their own interests, while ignoring the overall benefits of the supply chain. This behavior of both parties causes a double marginal effect, which will damage the overall profit of the supply chain.

### 4.2. Decentralized Decision Scenario With Cost-Sharing (DD)

Under this decision scenario, in order to encourage the retailer to actively carry out low-carbon promotion, the manufacturer will share a certain percentage of low-carbon promotion cost $\phi_{d}$ for the retailer. The decision sequence is: the manufacturer shares a certain percentage of low-carbon promotion cost for the retailer, and then, the retailer and manufacturer determine their own low-carbon promotion investment and emission reduction investment. First, we will keep the cost-sharing ratio fixed to calculate the equilibrium strategies of the manufacturer and retailer and then calculate the optimal cost-sharing ratio. Similarly, the objective functions of the manufacturer and retailer are as follows:

(7) $$J_{m}^{d}\left(G,R,t\right)=\underset{E}{\max}\int_{0}^{\infty }e^{-\rho t}\left[\pi_{mr}\left[\begin{array}{l} d_{r}+\left(\alpha_{r}+\beta_{r}\right)E\\ +kA+\mu G-\alpha_{r}R \end{array}\right]+\pi_{md}\left[\begin{array}{l} d_{d}+\left(\alpha_{d}+\beta_{d}\right)E\\ +\mu G+\xi kA-\alpha_{d}R \end{array}\right]-\frac{\eta_{m}E^{2}}{2}-\frac{\phi_{d}\eta_{r}A^{2}}{2}\right]dt$$

(8) $$J_{r}^{d}\left(G,R,t\right)=\underset{A}{\max}\int_{0}^{\infty }e^{-\rho t}\left[\pi_{rr}\left[d_{r}+\left(\alpha_{r}+\beta_{r}\right)E+kA+\mu G-\alpha_{r}R\right]-\frac{\left(1-\phi_{d}\right)\eta_{r}A^{2}}{2}\right]dt$$

**Theorem** **2.** *Under decentralized decision scenario with cost-sharing (DD), we can get the following results by solving the decision model:*

*(1)*  *The manufacturer’s optimal emission reduction investment is as follows*:
  $$E^{d*}=\frac{\pi_{mr}\left[\rho \left(\alpha_{r}+\beta_{r}\right)+\varepsilon \beta_{r}\right]+\pi_{md}\left[\rho \left(\alpha_{d}+\beta_{d}\right)+\varepsilon \beta_{d}\right]}{\eta_{m}\left(\rho +\varepsilon \right)}$$
*Based on the manufacturer’s low-carbon promotion cost-sharing ratio* $\phi_{d}$ *, the reaction function of the retailer’s optimal low-carbon promotion investment is as follows:*
  $$A^{d*}=\frac{\pi_{rr}\left[k\left(\rho +\sigma \right)+\lambda \mu \right]}{\left(1-\phi_{d}\right)\eta_{r}\left(\rho +\sigma \right)}$$
*(2)*  *The optimal evolution trajectory of low-carbon goodwill is as follows:*
  $$G^{d}\left(t\right)=\left(G_{0}-C^{d}\right)e^{-\sigma t}+C^{d},C^{d}=\frac{\lambda A^{d*}}{\sigma }$$
*(3)*  *The optimal evolution trajectory of the reference low-carbon level is as follows:*
  $$R^{d}\left(t\right)=\left(R_{0}-E^{d*}\right)e^{-\varepsilon t}+E^{d*}$$
*(4)*  *The present value functions of the manufacturer’s optimal profit and retailer’s optimal profit are as follows:*
  $$\begin{array}{l} J_{m}^{d*}=\frac{1}{\rho }\pi_{mr}\left(d_{r}+\beta_{r}E^{d*}+kA^{d*}\right)+\frac{1}{\sigma \left(\rho +\sigma \right)}\pi_{mr}\mu \left(G_{0}-\lambda A^{d*}\right)-\frac{1}{\left(\rho +\varepsilon \right)}\pi_{mr}\alpha_{r}\left(R_{0}-E^{d*}\right)-\frac{\eta_{m}E^{d*}{}^{2}}{2\rho }\\ +\frac{1}{\rho }\pi_{md}\left(d_{d}+\beta_{d}E^{d*}+\xi kA^{d*}\right)+\frac{1}{\sigma \left(\rho +\sigma \right)}\pi_{md}\mu \left(G_{0}-\lambda A^{d*}\right)-\frac{1}{\left(\rho +\varepsilon \right)}\pi_{md}\alpha_{d}\left(R_{0}-E^{d*}\right)-\frac{\phi_{d}^{*}\eta_{r}A^{d*}{}^{2}}{2\rho } \end{array}\;J_{r}^{d*}=\frac{1}{\rho }\pi_{rr}\left(d_{r}+\beta_{r}E^{d*}+kA^{d*}\right)+\frac{1}{\sigma \left(\rho +\sigma \right)}\pi_{rr}\mu \left(G_{0}-\lambda A^{d*}\right)-\frac{1}{\left(\rho +\varepsilon \right)}\pi_{rr}\alpha_{r}\left(R_{0}-E^{d*}\right)-\frac{\left(1-\phi_{d}^{*}\right)\eta_{r}A^{d*}{}^{2}}{2\rho }$$

The proof of Theorem 2 is similar to the proof of Theorem 1 and is omitted.

**Theorem** **3.** *Under decentralized decision scenario with cost-sharing (DD), the optimal low-carbon promotion cost-sharing ratio provided by the manufacturer is as follows:*

$$\phi_{d}^{*}=\{\begin{array}{ll} \frac{2A-B}{2A+B}, & 2A\geq B\\ 0,\; & 2A<B \end{array}$$

*where *$A=k\left(\rho +\sigma \right)\left(\pi_{mr}+\xi \pi_{md}\right)+\lambda \mu \left(\pi_{mr}+\pi_{md}\right)$*,*$B=\pi_{rr}\left[k\left(\rho +\sigma \right)+\lambda \mu \right]$.

The proof of Theorem 3 is easy to prove and is omitted.

Theorems 2 and 3 show that under the decentralized decision scenario with cost-sharing (DD), although manufacturers and retailers still make decisions based on their own interests; when manufacturers choose to share the cost of low-carbon promotion for retailers, retailers will actively carry out low-carbon promotion, which will help weaken the negative impact of the double marginal effect.

### 4.3. Decentralized Decision Scenario With Bilateral Cost-Sharing (DC) 

Under this decision scenario, not only does the manufacturer share a certain percentage of low-carbon promotion cost $\phi_{c}$ for the retailer, but the retailer also shares a certain percentage of emission reduction cost $\gamma_{c}$ for the manufacturer. The decision sequence is: the manufacturer shares a certain percentage of low-carbon promotion cost for the retailer, the retailer shares a certain percentage of emission reduction cost $\gamma_{c}$ for the manufacturer, and then the retailer and the manufacturer determine their own low-carbon promotion investment and emission reduction investment. Similarly, we will first keep the cost-sharing ratios fixed to calculate the equilibrium strategies of the manufacturer and retailer and then calculate the optimal cost-sharing ratios. At this time, the objective functions of the manufacturer and retailer are as follows:

(9) $$J_{m}^{c}\left(G,R,t\right)=\underset{E}{\max}\int_{0}^{\infty }e^{-\rho t}\left[\pi_{mr}\left[\begin{array}{l} d_{r}+\left(\alpha_{r}+\beta_{r}\right)E\\ +kA+\mu G-\alpha_{r}R \end{array}\right]+\pi_{md}\left[\begin{array}{l} d_{d}+\left(\alpha_{d}+\beta_{d}\right)E\\ +\mu G+\xi kA-\alpha_{d}R \end{array}\right]-\frac{\left(1-\gamma_{c}\right)\eta_{m}E^{2}}{2}-\frac{\phi_{c}\eta_{r}A^{2}}{2}\right]dt$$

(10) $$J_{r}^{c}\left(G,R,t\right)=\underset{A}{\max}\int_{0}^{\infty }e^{-\rho t}\left[\pi_{rr}\left[d_{r}+\left(\alpha_{r}+\beta_{r}\right)E+kA+\mu G-\alpha_{r}R\right]-\frac{\left(1-\phi_{d}\right)\eta_{r}A^{2}}{2}-\frac{\gamma_{c}\eta_{m}E^{2}}{2}\right]dt$$

**Theorem** **4.** *Under decentralized decision scenario with bilateral cost-sharing (DC), we can get the following results by solving the decision model:*

*(1)*  *Based on the manufacturer’s low-carbon promotion cost-sharing ratio* $\phi_{c}$ *and the retailer’s emission reduction cost-sharing ratio* $\gamma_{c}$ *, the reaction functions of the manufacturer’s optimal emission reduction investment and the retailer’s optimal low-carbon promotion investment are as follows:*
  $$\left\{ \begin{array}{l} E^{c*}=\frac{\pi_{mr}\left[\rho \left(\alpha_{r}+\beta_{r}\right)+\varepsilon \beta_{r}\right]+\pi_{md}\left[\rho \left(\alpha_{d}+\beta_{d}\right)+\varepsilon \beta_{d}\right]}{\left(1-\gamma_{c}\right)\eta_{m}\left(\rho +\varepsilon \right)}\\ A^{c*}=\frac{\pi_{rr}\left[k\left(\rho +\sigma \right)+\lambda \mu \right]}{\left(1-\phi_{c}\right)\eta_{r}\left(\rho +\sigma \right)} \end{array}\right.$$
*(2)*  *The optimal evolution trajectory of low-carbon goodwill is as follows:*
  $$G^{c}\left(t\right)=\left(G_{0}-C^{c}\right)e^{-\sigma t}+C^{c},C^{c}=\frac{\lambda A^{c*}}{\sigma }$$
*(3)*  *The optimal evolution trajectory of reference low-carbon level is as follows:*
  $$R^{c}\left(t\right)=\left(R_{0}-E^{c*}\right)e^{-\varepsilon t}+E^{c*}$$
*(4)*  *The present value functions of the manufacturer’s optimal profit and retailer’s optimal profit are as follows:*
  $$\begin{array}{l} J_{m}^{c*}=\frac{1}{\rho }\pi_{mr}\left(d_{r}+\beta_{r}E^{c*}+kA^{c*}\right)+\frac{1}{\sigma \left(\rho +\sigma \right)}\pi_{mr}\mu \left(G_{0}-\lambda A^{c*}\right)-\frac{1}{\left(\rho +\varepsilon \right)}\pi_{mr}\alpha_{r}\left(R_{0}-E^{c*}\right)-\frac{\left(1-\gamma_{c}^{*}\right)\eta_{m}E^{c*}{}^{2}}{2\rho }\\ +\frac{1}{\rho }\pi_{md}\left(d_{d}+\beta_{d}E^{c*}+\xi kA^{c*}\right)+\frac{1}{\sigma \left(\rho +\sigma \right)}\pi_{md}\mu \left(G_{0}-\lambda A^{c*}\right)-\frac{1}{\left(\rho +\varepsilon \right)}\pi_{md}\alpha_{d}\left(R_{0}-E^{c*}\right)-\frac{\phi_{c}^{*}\eta_{r}A^{c*}{}^{2}}{2\rho } \end{array}\;\begin{array}{l} J_{r}^{c*}=\frac{1}{\rho }\pi_{rr}\left(d_{r}+\beta_{r}E^{c*}+kA^{c*}\right)+\frac{1}{\sigma \left(\rho +\sigma \right)}\pi_{rr}\mu \left(G_{0}-\lambda A^{c*}\right)-\frac{1}{\left(\rho +\varepsilon \right)}\pi_{rr}\alpha_{r}\left(R_{0}-E^{c*}\right)-\frac{\gamma_{c}^{*}\eta_{m}E^{c*}{}^{2}}{2\rho }\\ -\frac{\left(1-\phi_{c}^{*}\right)\eta_{r}A^{c*}{}^{2}}{2\rho } \end{array}$$

**Theorem** **5.** *Under decentralized decision scenario with bilateral cost-sharing (DC), the optimal low-carbon promotion cost-sharing ratio provided by the manufacturer is as follows: *

$$\phi_{c}^{*}=\{\begin{array}{ll} \frac{2A-B}{2A+B}, & 2A\geq B\\ 0,\; & 2A<B \end{array}\;$$

*where*$A=k\left(\rho +\sigma \right)\left(\pi_{mr}+\xi \pi_{md}\right)+\lambda \mu \left(\pi_{mr}+\pi_{md}\right)$*, *$B=\pi_{rr}\left[k\left(\rho +\sigma \right)+\lambda \mu \right]$.

*The optimal emission reduction cost-sharing ratio provided by the retailer is as follows:*

$$\gamma_{c}^{*}=\{\begin{array}{ll} \frac{2C-D}{2C+D}, & 2C\geq D\\ 0, & 2C<D \end{array}$$

*where*$C=\pi_{rr}\left[\rho \left(\alpha_{r}+\beta_{r}\right)+\varepsilon \beta_{r}\right]$*, *$D=\pi_{mr}\left[\rho \left(\alpha_{r}+\beta_{r}\right)+\varepsilon \beta_{r}\right]+\pi_{md}\left[\rho \left(\alpha_{d}+\beta_{d}\right)+\varepsilon \beta_{d}\right]$.

The proof of Theorems 4 and 5 is similar to the previous proof and is omitted.

Theorems 4 and 5 show that under the decentralized decision scenario with bilateral cost-sharing (DC), although manufacturers and retailers still make decisions based on their own interests, when manufacturers choose to share the cost of low-carbon promotion for retailers, and meanwhile, retailers also choose to share manufacturers’ abatement costs, this will help to incentivize both parties to continuously increase emissions reduction investment and will further help weaken the negative impact of the double marginal effect.

### 4.4. Centralized Decision Scenario(CC)

Under the centralized decision scenario, the manufacturer and retailer have reached a cooperation agreement. To maximize the overall profit of the supply chain, the two parties will jointly determine the emission reduction level and low-carbon promotion level. In addition, the present value of the overall profit of the supply chain will be used as the supply chain coordination benchmarking. At this time, the objective function of the supply chain is as follows:

(11) $$J_{sc}^{cc}\left(G,R,t\right)=\underset{E}{\max}\int_{0}^{\infty }e^{-\rho t}\left[\left(\pi_{mr}+\pi_{rr}\right)\left[\begin{array}{l} d_{r}+\left(\alpha_{r}+\beta_{r}\right)E\\ +kA+\mu G-\alpha_{r}R \end{array}\right]+\pi_{md}\left[\begin{array}{l} d_{d}+\left(\alpha_{d}+\beta_{d}\right)E\\ +\mu G+\xi kA-\alpha_{d}R \end{array}\right]-\frac{\eta_{m}E^{2}}{2}-\frac{\eta_{r}A^{2}}{2}\right]dt$$

**Theorem** **6.** *Under centralized decision scenario (CC), we can get the following results by solving the decision model:*

*(1)*  *Differential game equilibrium strategies of the manufacturer and retailer are as follows:*
  $$\left\{ \begin{array}{l} E^{cc*}=\frac{\left(\pi_{mr}+\pi_{rr}\right)\left[\rho \left(\alpha_{r}+\beta_{r}\right)+\varepsilon \beta_{r}\right]+\pi_{md}\left[\rho \left(\alpha_{d}+\beta_{d}\right)+\varepsilon \beta_{d}\right]}{\eta_{m}\left(\rho +\varepsilon \right)}\\ A^{cc*}=\frac{\left[\left(\pi_{mr}+\pi_{rr}\right)k+\pi_{md}\xi k\right]\left(\rho +\sigma \right)+\lambda \mu \left(\pi_{mr}+\pi_{rr}+\pi_{md}\right)}{\eta_{r}\left(\rho +\sigma \right)} \end{array}\right.$$
*(2)*  *The optimal evolution trajectory of low-carbon goodwill is as follows:*
  $$G^{cc}\left(t\right)=\left(G_{0}-C^{cc}\right)e^{-\sigma t}+C^{cc},C^{cc}=\frac{\lambda A^{cc*}}{\sigma }$$
*(3)*  *The optimal evolution trajectory of the reference low-carbon level is as follows:*
  $$R^{cc}\left(t\right)=\left(R_{0}-E^{cc*}\right)e^{-\varepsilon t}+E^{cc*}$$
*(4)*  *The present value function of the optimal overall profit of the supply chain is as follows:*
  $$\begin{array}{l} J_{sc}^{cc*}=\frac{1}{\rho }\left(\pi_{mr}+\pi_{rr}\right)\left(d_{r}+\beta_{r}E^{cc*}+kA^{cc*}\right)+\frac{1}{\sigma \left(\rho +\sigma \right)}\left(\pi_{mr}+\pi_{rr}+\pi_{md}\right)\mu \left(G_{0}-\lambda A^{cc*}\right)-\frac{\eta_{r}A^{cc*}{}^{2}}{2\rho }\\ -\frac{1}{\left(\rho +\varepsilon \right)}\left(\pi_{mr}+\pi_{rr}\right)\alpha_{r}\left(R_{0}-E^{cc*}\right)+\frac{1}{\rho }\pi_{md}\left(d_{d}+\beta_{d}E^{cc*}+\xi kA^{cc*}\right)-\frac{1}{\left(\rho +\varepsilon \right)}\pi_{md}\alpha_{d}\left(R_{0}-E^{cc*}\right)\\ -\frac{\eta_{m}E^{cc*}{}^{2}}{2\rho } \end{array}$$

The proof of Theorem 6 is similar to the previous proof and is omitted.

Theorem 6 shows that under the centralized decision scenario (CC), at this time, manufacturers and retailers will make decisions from the perspective of the overall profits of the supply chain. Manufacturers’ investment in emission reduction and retailers’ low-carbon publicity investment will both reach the maximum. The two parties actively communicate and cooperate to improve the overall operating efficiency of the supply chain and the overall efficiency of the supply chain.

## 5. Analysis and Discussion

Through comparative static analysis of key parameters under the three decentralized decision scenarios (see Table 3 for details), Corollary 1 can be obtained.

**Corollary** **1.**

*(1)*  *Under the three decentralized decision scenarios, the manufacturer’s emission reduction investment and stable reference low-carbon level have a positive relationship with the marginal profit of the two channels and discount rate and have a negative relationship with the emission reduction cost coefficient and memory parameter.*
*(2)*  *Under the three decentralized decision scenarios, the retailer’s low-carbon promotion investment and stable low-carbon goodwill level have a positive relationship with its marginal profit and a negative relationship with the discount rate and low-carbon promotion cost coefficient.*

It can be seen from Corollary 1 that the manufacturer’s emission reduction investment is positively correlated with its marginal profit obtained in the two channels, so although emission reduction brings a certain cost burden to the manufacturer, the increase in product sales caused by emission reduction and the channel marginal profit obtained from product sales will still actively promote manufacturers to invest in emission reduction. In addition, in order to encourage manufacturers to actively invest in emission reduction, on the one hand, manufacturers should improve emission reduction efficiency and reduce emission reduction cost by updating production equipment and production materials and upgrading emission reduction technologies. On the other hand, manufacturers should also actively cooperate with other members of the supply chain to share the cost of emission reduction.

In addition, similar to the manufacturer’s investment in reducing carbon emissions, the retailer’s low-carbon promotion investment is also positively correlated with its marginal profit and negatively correlated with its low-carbon promotion costs. Therefore, in order to encourage retailers to actively carry out low-carbon publicity, they should actively change publicity methods, improve publicity efficiency, and reduce publicity costs. On the other hand, it is not necessary to blindly pursue low-carbon publicity investment but also pay attention to the effect of low-carbon publicity and use low-carbon publicity investment to drive the accumulation of low-carbon goodwill and ultimately enhance consumers’ loyalty to the low-carbon product brand.

Through comparative static analysis of key parameters under the centralized decision scenario (see Table 4 for details), Corollary 2 can be obtained.

**Corollary** **2.** *Compared with the three decentralized decision scenarios, the manufacturer’s emission reduction investment and stable reference low-carbon level under the centralized decision scenario are not only positively related to the marginal profit of the two channels, but also positively related to the retailer’s marginal profit. The retailer’s low-carbon publicity investment and stable low-carbon goodwill level are not only positively related to its own marginal profit but are also positively related to the manufacturer’s marginal profit of the two channels and the indirect spillover effect coefficient.*

It can be seen from Corollary 2 that compared with decentralized decision scenarios, whether the manufacturer make its own emission reduction investment, or the retailer make its own low-carbon publicity investment, both of them will pay more attention to the overall profit of the supply chain. For example, when the retailer determines low-carbon publicity investment under the centralized decision scenario, it is not only concerned with the marginal profit of its own retail channel but with the sum of its own retail channel marginal profit and the manufacturer’s marginal profit of two channels.

**Corollary** **3.** *When manufacturers and retailers are willing to share emission reduction costs for each other, we have:*

$$E^{cc*}>E^{c*}>E^{d*}=E^{n*};\;A^{cc*}>A^{c*}=A^{d*}>A^{n*};\;J_{m}^{c*}>J_{m}^{d*}>J_{m}^{n*}\;J_{r}^{c*}>J_{r}^{d*}>J_{r}^{n*};\;J_{sc}^{cc*}>J_{sc}^{c*}>J_{sc}^{d*}>J_{sc}^{n*};$$

Corollary 3 shows that compared with decentralized decision scenario, the manufacturer’s emission reduction level and the retailer’s low-carbon publicity level under the centralized decision scenario are higher. The manufacturer’s low-carbon publicity cost sharing for the retailer will not affect the manufacturer’s emission reduction level, but it will effectively improve the low-carbon publicity level of the retailer. Similarly, when the retailer provides the manufacturer with emission reduction cost sharing, it will not affect the low-carbon publicity level of the retailer, but it will effectively increase the emission reduction level of the manufacturer. Sharing emission reduction costs with each other can effectively improve the profit of both parties. The overall profit of the supply chain is the largest under centralized decision making. Sharing emission reduction costs with each other can effectively increase the overall profit of the supply chain.

**Corollary** **4.**

*(1)*  *when*2A≥B*, then*$\frac{\partial \phi_{d}^{*}}{\partial \pi_{mr}}>0$*,*$\frac{\partial \phi_{d}^{*}}{\partial \pi_{md}}>0$*,*$\frac{\partial \phi_{d}^{*}}{\partial \pi_{rr}}<0$.
*(2)*  *when*2C≥D*, then*$\frac{\partial \gamma_{c}^{*}}{\partial \pi_{rr}}>0$*,*$\frac{\partial \gamma_{c}^{*}}{\partial \pi_{mr}}>0$*,*$\frac{\partial \gamma_{c}^{*}}{\partial \pi_{md}}<0$.

Corollary 4 shows that when the manufacturer provides the retailer with low-carbon publicity cost sharing, the cost-sharing ratio will increase with the increase in the manufacturer’ marginal profit of the two channels but will decrease with the increase in the retailer’s marginal profit. Similarly, when the retailer provides the manufacturer with emission reduction cost sharing, the cost-sharing ratio will also increase with the increase in the retailer’s marginal profit but will decrease with the increase in the manufacturer’s marginal profit of the two channels. The above phenomenon is consistent with reality. When the channel profit of the manufacturer increases, the manufacturer will naturally strengthen its motivation for the retailer’s low-carbon promotion, and on the contrary, it will weaken its willingness to share the low-carbon promotion cost for the retailer. The increase in the retailer’s marginal profit means that the retailer will gain stronger economic power, and it will have more resources to invest in low-carbon publicity. In other words, when facing the same low-carbon publicity investment, even if the manufacturer reduces its economic support, the retailer still has the strength to carry out low-carbon publicity activities. This is also true when the retailer shares the emission reduction cost with the manufacturer.

**Corollary** **5.**

*(1)*  $\frac{\partial \left(A^{cc*}-A^{n*}\right)}{\partial \pi_{mr}}>0$*,*$\frac{\partial \left(A^{cc*}-A^{n*}\right)}{\partial \pi_{md}}>0$*,*$\frac{\partial \left(A^{cc*}-A^{n*}\right)}{\partial k}>0$*,*$\frac{\partial \left(A^{cc*}-A^{n*}\right)}{\partial \lambda }>0$*,*$\frac{\partial \left(A^{cc*}-A^{n*}\right)}{\partial \mu }>0$.
*(2)*  $\frac{\partial \left(E^{cc*}-E^{n*}\right)}{\partial \pi_{rr}}>0$*,*$\frac{\partial \left(E^{cc*}-E^{n*}\right)}{\partial \alpha_{r}}>0$*,*$\frac{\partial \left(E^{cc*}-E^{n*}\right)}{\partial \beta_{r}}>0$.

Corollary 5 shows that the difference between the retailer’s low-carbon publicity level under the decentralized decision scenario and centralized decision scenario will increase with the increase in the manufacturer’s marginal profits of the two channels. The difference in the manufacturer’s emission reduction levels under the decentralized decision scenario and centralized decision scenario will increase as the retailer’s marginal profit increases. The above situation means that the greater the manufacturer’s marginal profits of the two channels, the greater the value of the supply chain members’ cooperation to the retailer’s low-carbon publicity investment. The greater the retailer’s marginal profit, the greater the value of the supply chain members’ cooperation to the manufacturer’s emission reduction investment. The above phenomenon is consistent with reality, because the cooperation between supply chain members is a process in which members absorb each other’s advantages to enhance their own strength, that is, the stronger the opponent is, the more the value of cooperation can be displayed.

**Corollary** **6.** $\frac{\partial^{2}E^{n*}}{\partial \varepsilon^{2}}>0$, $\frac{\partial^{2}E^{n*}}{\partial \varepsilon \partial \pi_{mr}}<0$, $\frac{\partial^{2}E^{n*}}{\partial \varepsilon \partial \pi_{md}}<0$,$\frac{\partial^{2}E^{n*}}{\partial \varepsilon \partial \alpha_{r}}<0$, $\frac{\partial^{2}E^{n*}}{\partial \varepsilon \partial \alpha_{d}}<0$.

Corollary 6 shows that with the gradual increase in the memory parameter, the decrease rate of the manufacturer’s emission reduction investment with the increase in the memory parameter will be lower. While with the gradual increase in the manufacturer’s marginal profit of the two channels, the decrease rate of the manufacturer’s emission reduction investment with the increase in the memory parameter will be higher. Similarly, with the gradual increase in the influence coefficient of consumers’ reference low-carbon effect on the demand of online and offline channels, the decrease rate of the manufacturer’s emission reduction investment with the increase in the memory parameter will also be higher. 

## 6. Supply Chain Coordination Contract (CS)

Through the above comparison and analysis, it can be seen that compared to various decentralized decision scenarios, the optimal decisions and profit of the supply chain under the centralized decision scenario are greater. Therefore, it is necessary to design an appropriate coordination contract to allocate the overall profit of the supply chain under the centralized decision scenario. Here, we will coordinate the supply chain by designing a cost-sharing coordination contract. In the cost-sharing coordination contract, both two parties will adopt the optimal decisions under the centralized decision scenario. At the same time, the manufacturer as the leader will distribute the overall profit of the supply chain under the centralized decision scenario through a reasonable low-carbon publicity cost-sharing ratio. Through the above analysis, the present value functions of the manufacturer’s profit and the retailer’s profit are as follows:

(12) $$\begin{array}{l} J_{m}^{cs*}=\frac{1}{\rho }\pi_{mr}\left(d_{r}+\beta_{r}E^{cc*}+kA^{cc*}\right)+\frac{1}{\sigma \left(\rho +\sigma \right)}\pi_{mr}\mu \left(G_{0}-\lambda A^{cc*}\right)-\frac{1}{\left(\rho +\varepsilon \right)}\pi_{mr}\alpha_{r}\left(R_{0}-E^{cc*}\right)-\frac{\eta_{m}E^{cc*}{}^{2}}{2\rho }\\ +\frac{1}{\rho }\pi_{md}\left(d_{d}+\beta_{d}E^{cc*}+\xi kA^{cc*}\right)+\frac{1}{\sigma \left(\rho +\sigma \right)}\pi_{md}\mu \left(G_{0}-\lambda A^{cc*}\right)-\frac{\phi_{cs}^{*}\eta_{r}A^{cc*}{}^{2}}{2\rho }-\frac{1}{\left(\rho +\varepsilon \right)}\pi_{md}\alpha_{d}\left(R_{0}-E^{cc*}\right) \end{array}$$

(13) $$\begin{array}{l} J_{r}^{cs*}=\frac{1}{\rho }\pi_{rr}\left(d_{r}+\beta_{r}E^{cc*}+kA^{cc*}\right)+\frac{1}{\sigma \left(\rho +\sigma \right)}\pi_{rr}\mu \left(G_{0}-\lambda A^{cc*}\right)-\frac{1}{\left(\rho +\varepsilon \right)}\pi_{rr}\alpha_{r}\left(R_{0}-E^{cc*}\right)\\ -\frac{\left(1-\phi_{cs}^{*}\right)\eta_{r}A^{cc*}{}^{2}}{2\rho } \end{array}$$

At this time, we make the sum of the profits of the manufacturer and the retailer equal to the overall profit of the supply chain under the centralized decision scenario, and we can obtain Formula (14).

(14) $$J_{m}^{cs*}+J_{r}^{cs*}=J_{sc}^{cc*}$$

The necessary condition for realizing supply chain coordination is that when the low-carbon publicity cost ratio $\phi_{cs}^{\max}\in \left[0,1\right]$, the manufacturer’s profit cannot be lower than the manufacturer’s optimal profit under the decentralized decision scenario, that is:

(15) $$J_{m}^{cs*}\geq J_{m}^{d*},0\leq \phi_{cs}^{\max}\leq 1$$

Through Formula (15), the maximum low-carbon publicity cost-sharing ratio that the manufacturer is willing to bear for the retailer is as follows:

(16) $$\phi_{cs}^{\max}=\frac{2\rho \left\{\begin{array}{l} \left[\frac{1}{\rho }\pi_{mr}\beta_{r}+\frac{1}{\left(\rho +\varepsilon \right)}\pi_{mr}\alpha_{r}+\frac{1}{\rho }\pi_{md}\beta_{d}+\frac{1}{\left(\rho +\varepsilon \right)}\pi_{md}\alpha_{d}\right]\left(E^{cc*}-E^{d*}\right)+\frac{\eta_{m}}{2\rho }\left(E^{d*}{}^{2}-E^{cc*}{}^{2}\right)\\ +\left[\frac{1}{\rho }\pi_{mr}k-\frac{1}{\sigma \left(\rho +\sigma \right)}\pi_{mr}\mu \lambda +\frac{1}{\rho }\pi_{md}\xi k-\frac{1}{\sigma \left(\rho +\sigma \right)}\pi_{md}\mu \lambda \right]\left(A^{cc*}-A^{d*}\right)+\frac{\phi_{d}^{*}\eta_{r}A^{d*}{}^{2}}{2\rho } \end{array}\right\}}{\eta_{r}A^{cc*}{}^{2}}$$

Similarly, the necessary coordination condition that the retailer is willing to accept is that the low-carbon publicity cost ratio $\phi_{cs}^{\min}\in \left[0,1\right]$, the retailer’s profit cannot be lower than its optimal profit under the decentralized decision scenario, that is:

(17) $$J_{r}^{cs*}\geq J_{r}^{d*},0\leq \phi_{cs}^{\min}\leq 1$$

Through Formula (17), the minimum low-carbon publicity cost-sharing ratio that the retailer is willing to accept is as follows:

(18) $$\phi_{cs}^{\min}=1-\frac{2\rho \left\{\begin{array}{l} \left[\frac{1}{\rho }\pi_{rr}\beta_{r}+\frac{1}{\left(\rho +\varepsilon \right)}\pi_{rr}\alpha_{r}\right]\left(E^{cc*}-E^{d*}\right)+\left[\frac{1}{\rho }\pi_{rr}k-\frac{1}{\sigma \left(\rho +\sigma \right)}\pi_{rr}\mu \lambda \right]\left(A^{cc*}-A^{d*}\right)\\ +\frac{\left(1-\phi_{d}^{*}\right)\eta_{r}A^{d*}{}^{2}}{2\rho } \end{array}\right\}}{\eta_{r}A^{cc*}{}^{2}}$$

**Theorem** **7.** *When the manufacturer’s low-carbon publicity cost-sharing ratio for the retailer meets* $\phi_{cs}\in \left[\phi_{cs}^{\min},\phi_{cs}^{\max}\right]$ *,* $\phi_{cs}^{\min}\in \left[0,1\right]$ *, and* $\phi_{cs}^{\max}\in \left[0,1\right]$ *, this low-carbon publicity cost-sharing mechanism can achieve supply chain coordination, and the value of the low-carbon publicity cost-sharing ratio* $\phi_{cs}$ *depends on the bargaining power of the manufacturer and retailer.*

Theorem 7 shows that when the low-carbon publicity cost-sharing ratio provided by the manufacturer to the retailer is within a reasonable range, the coordination of the supply chain can be achieved, the ratio is not a fixed value, and its value can be negotiated by both parties.

## 7. Numerical Analysis

Considering the complexity of the solutions, some analysis is difficult to carry out. In this section, we will use numerical examples to further verify the previous research conclusions and carry out sensitivity analysis of the equilibrium strategies and profits of the manufacturer and retailer under the different decision scenarios. Referring to the previous related literature and combining the specific background of our research, we set the related parameters involved in this paper as follows: $\pi_{mr}=1$, $\pi_{md}=1$, $\pi_{rr}=0.5$, $\eta_{m}=1$, $\eta_{r}=1$, ρ=0.1, ε=2, λ=0.1, $\alpha_{r}=0.2$, $\alpha_{d}=0.1$, $\beta_{r}=0.8$, $\beta_{d}=0.8$, k=0.4, μ=0.1, σ=1, ξ=0.5, $d_{d}=2$, $d_{r}=2$, $R_{0}=3$, $G_{0}=2$. To ensure the accuracy of the analysis, when performing sensitivity analysis on certain parameters, we ensured that the values of other parameters remain fixed.

**Figure 1:**
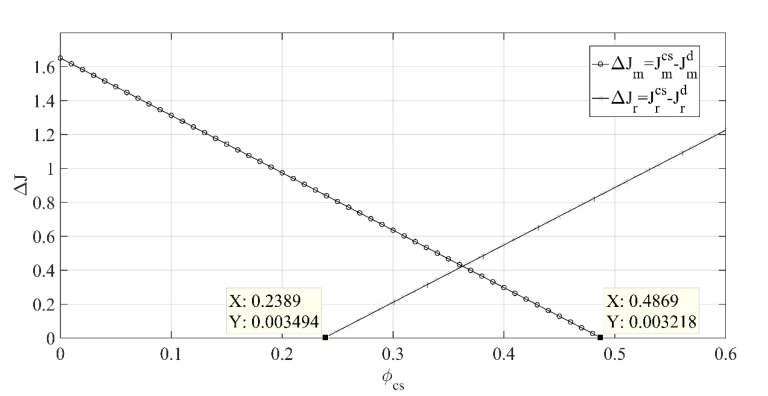
The effect of $\phi_{cs}$ on the present value of profits.

(1) The impact of low-carbon publicity cost-sharing ratio $\phi_{cs}$ on the difference in the present value of profit under the coordination mechanism and decentralized decision scenario with cost sharing is shown in Figure 1.

Figure 1 shows that with the increase in low-carbon publicity cost-sharing ratio $\phi_{cs}$, the difference of the manufacturer’s optimal profit under two decision scenarios will decrease, while the difference of the retailer’s optimal profit under two decision scenarios will increase. When the cost-sharing ratio $\phi_{cs}\in \left(0,0.5\right)$, the manufacturer’s optimal profit under the coordination mechanism scenario will not be lower than that under the cost-sharing decentralized decision scenario. When the cost-sharing ratio $\phi_{cs}\in \left(0.24,1\right)$, the retailer’s optimal profit under the coordination mechanism scenario will not be lower than that under the cost-sharing decentralized decision scenario. Therefore, when the low-carbon publicity cost-sharing ratio $\phi_{cs}\in \left(0.24,0.5\right)$, the two parties’ profits under the coordination mechanism scenario will not be lower than that under the cost-sharing decentralized decision scenario, and the coordination mechanism is established.

The above conclusion shows that in real life, both parties should consider from many aspects, communicate and negotiate more, and determine the appropriate share ratio under the condition that the interests of both parties are not harmed to ensure that the two parties can carry out effective cooperation.

**Figure 2:**
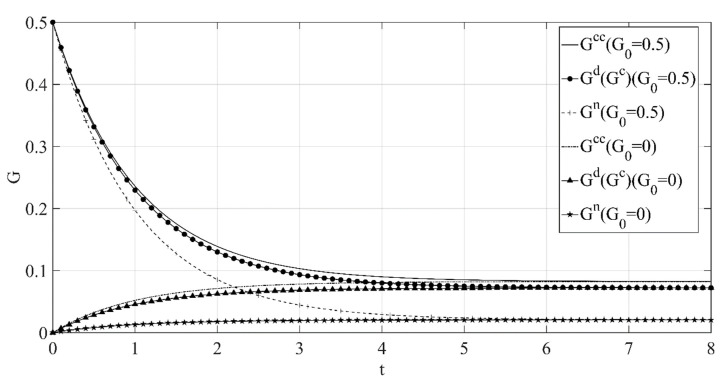
The optimal path of low-carbon goodwill over time.

(2) The optimal path of low-carbon goodwill over time under the different initial low-carbon goodwill levels is shown in Figure 2.

Figure 2 shows that when the initial low-carbon goodwill is low, as time goes by, the low-carbon goodwill under the different decision scenarios will increase, while when the initial low-carbon goodwill is high, as time goes by, the low-carbon goodwill under the different decision scenarios will decrease. Even if the initial low-carbon goodwill level will affect its path over time, when reaching a certain period, the low-carbon goodwill will stabilize at the same level under the same decision scenarios, and the stable value is positively correlated with the retailer’s low-carbon publicity investment, so it is not difficult to find that the stable low-carbon goodwill level under the centralized decision scenario is the highest, and the stable low-carbon goodwill level under the decision scenario without cost sharing is the lowest. 

**Figure 3:**
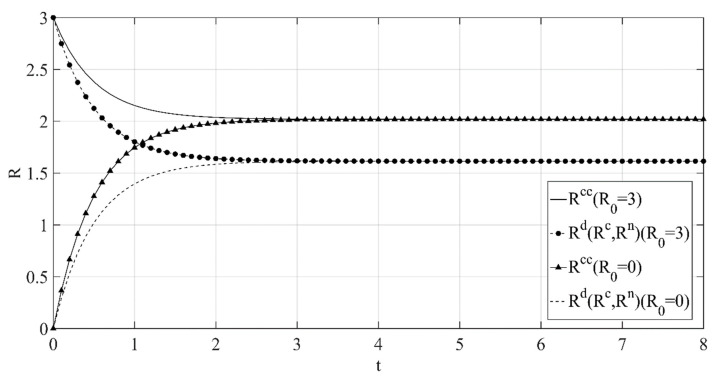
The optimal path of reference low-carbon level over time.

(3) The optimal path of reference low-carbon level over time under the different initial reference low-carbon levels is shown in Figure 3.

Similar to low-carbon goodwill, Figure 3 shows that when the initial reference low-carbon level is low, the reference low-carbon level will increase over time under the different decision scenarios, while when the initial reference low-carbon level is high, the reference low-carbon level will decrease over time. Similarly, even though the initial reference low-carbon level will affect its path over time, when reaching a certain period of time, the reference low-carbon level will stabilize at the same level under the same decision scenario, and this stable value is positively related to the manufacturer’s emission reduction investment, so it is not difficult to find that the stable reference low-carbon level is the highest under the centralized decision scenario compared with the reference low-carbon levels under other decentralized decision scenarios. 

(4) Sensitivity analysis on key parameters of the manufacturer’s emission reduction investment.

**Figure 4:**
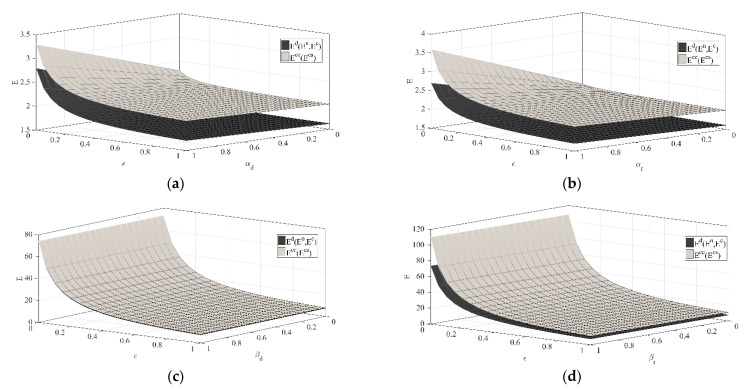
The impact of effectiveness parameters on the manufacturer’s emission reduction investment. (**a**) The effect of $\alpha_{r}$ and ε. (**b**) The effect of $\alpha_{d}$ and ε. (**c**) The effect of $\beta_{r}$ and ε. (**d**) The effect of $\beta_{d}$ and ε.

Figure 4a,b shows that with the increase in memory parameter ε, the manufacturer’s emission reduction investment under each decision scenario will decrease, and as $\alpha_{r}$, $\alpha_{d}$ increase, the manufacturer’s emission reduction investment will increase. In addition, it can be clearly seen that when $\alpha_{r}$ or $\alpha_{d}$ is larger, the decrease rate of the manufacturer’s emission reduction investment with the increase in the memory parameter ε is also higher. While when ε is larger, the increase rate of the manufacturer’s emission reduction investment with the increase in $\alpha_{r}$ or $\alpha_{d}$ is lower. The above phenomenon shows that when consumers are more sensitive to reference low-carbon effect, and they tend to have a long-term memory of products’ emission reductions, this will effectively promote manufacturers to increase emission reduction efforts. Conversely, if consumers are insensitive or less sensitive to reference low-carbon effect, and they tend to have a short-term memory of products’ emission reductions, this cannot encourage manufacturers to actively reduce emissions.

Figure 4c,d shows that with the increase in memory parameter ε, the manufacturer’s emission reduction investment under each decision scenario will decrease, and as $\beta_{r}$, $\beta_{d}$ increase, the manufacturer’s emission reduction investment will increase. In addition, it can also be clearly seen that when $\beta_{r}$ or $\beta_{d}$ is larger, the decrease rate of the manufacturer’s emission reduction investment with the increase in the memory parameter ε is also higher. While when ε is larger, the increase rate of the manufacturer’s emission reduction investment with the increase in $\beta_{r}$ or $\beta_{d}$ is lower. The above phenomenon shows that when consumers are more sensitive to products’ emission reductions, and they tend to have a long-term memory of products’ emission reductions, this will effectively promote manufacturers to increase emission reduction efforts. Conversely, if consumers are insensitive or less sensitive to products’ emission reductions, and they tend to have a short-term memory of products’ emission reductions, this cannot encourage manufacturers to actively reduce emissions.

(5) Sensitivity analysis on key parameters of the retailer’s low-carbon publicity investment.

**Figure 5:**
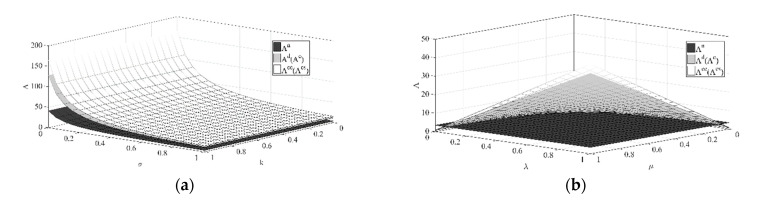
The impact of effectiveness parameters on the retailer’s low-carbon publicity investment. (**a**) The effect of σ and k. (**b**) The effect of λ and μ.

It can be seen from Figure 5a that the retailer’s low-carbon publicity investment under different decision scenarios will decrease with the increase in σ but will increase with the increase in k. In addition, it can be clearly seen that compared with k, the impact degree of σ is greater. In particular, when k is larger, the decrease rate of the retailer’s low-carbon publicity investment with the increase in σ is also higher. While when σ is larger, the increase rate of the retailer’s low-carbon publicity investment with the increase in k is lower. The above phenomenon shows that compared with the direct promotion of low-carbon publicity investment to low-carbon product sales, retailers pay more attention to the establishment of low-carbon product brands. When consumers in the market are more sensitive to the product promotion level, and when they purchase low-carbon products, they pay more attention to low-carbon product brands, this will effectively promote retailers to increase low-carbon publicity investment. Conversely, if consumers are insensitive or less sensitive to the low-carbon publicity level, or they do not pay attention to low-carbon product brands when buying low-carbon products, this cannot promote retailers to actively conduct low-carbon publicity.

Figure 5b shows that the retailer’s low-carbon publicity investment under different decision scenarios will increase with the increase in λ and will also increase with the increase in μ. In addition, it can be clearly seen that compared with k, the impact degree of σ is greater. Especially when λ and μ are both larger, the retailer’s low-carbon publicity investment will also reach a higher level under each decision scenario. The above phenomenon shows that if the retailer’s low-carbon publicity investment can more effectively help establish low-carbon product brands, and consumers in the market pay more attention to low-carbon product brands when buying low-carbon products, it will effectively promote retailers to increase low-carbon publicity efforts. Conversely, if the retailer’s low-carbon publicity investment has little effect on the establishment of low-carbon product brands, and consumers do not pay attention to low-carbon product brands when buying low-carbon products, it will not be able to encourage retailers to actively conduct low-carbon publicity.

(6) Sensitivity analysis on key parameters of the manufacturer’s profit and retailer’s profit.

**Figure 6:**
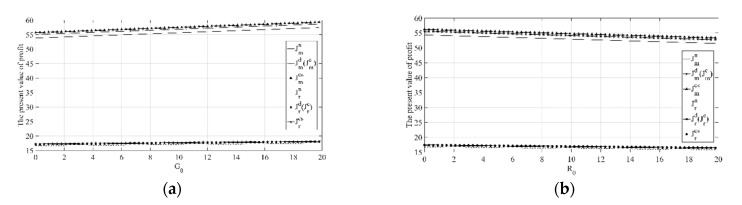
The impact of initial low-carbon goodwill and reference low-carbon level on profits. (**a**) The effect of $G_{0}$ on profits. (**b**) The effect of $R_{0}$ on profits.

It can be seen from Figure 6a that the profits of the manufacturer and retailer under different decision scenarios both increase with the increase in the initial low-carbon goodwill, that is, a higher initial low-carbon goodwill level will have a positive effect on the both parties’ profits. This is consistent with the reality. When the initial goodwill level of low-carbon products is very high, that is, consumers in the market have a high recognition degree of the low-carbon products from the beginning, then the manufacturer can easily meet the requirements of consumers through a certain amount of emission reduction investment, thereby having a positive effect on the demand of the product, and ultimately bringing higher profits to both parties. At the same time, as the initial goodwill of the low-carbon product continues to increase, that is, consumers’ initial recognition degree of the low-carbon product is getting higher and higher, then the manufacturer will be more able to meet the requirements of consumers through a certain amount of emission reduction investment, thereby having a positive effect on the demand of the product, and ultimately bringing higher profits to both parties.

It can be seen from Figure 6b that the profits of the manufacturer and retailer will decrease with the increase in the initial reference low-carbon level. That is to say, an excessively high initial reference low-carbon level will have a detrimental effect on the profits of both parties. This is because when the consumer’s initial reference low-carbon level is low, that is, consumers in the market have lower requirements for products’ carbon emission reductions, the manufacturer can easily meet the requirements of consumers, thereby having a positive effect on the demand of the product, and ultimately bringing higher profits to both parties. When the consumer’s initial reference low-carbon level is high, that is, consumers on the market have higher requirements for products’ carbon emission reductions, at this time, the manufacturer will find it difficult to satisfy consumers’ requirements through certain emission reduction investment, thereby having a negative impact on the demand of the product, and ultimately leading to a decline in the profits of both parties.

**Figure 7:**
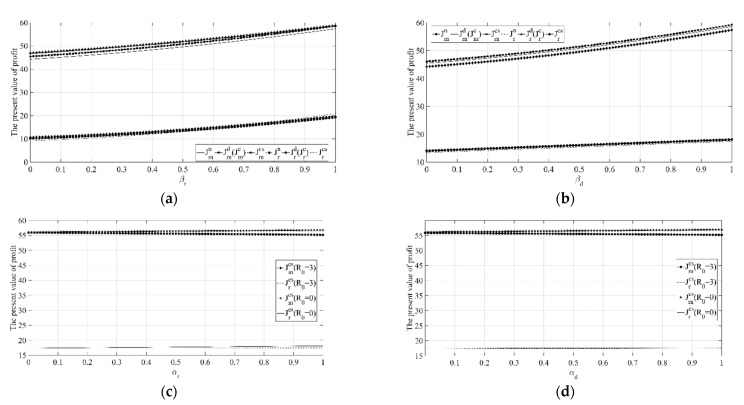
The impact of effectiveness parameters on profits. (**a**) The effect of $\beta_{r}$ on profits. (**b**) The effect of $\beta_{d}$ on profits. (**c**) The effect of $\alpha_{r}$ on profits. (**d**) The effect of $\alpha_{d}$ on profits.

It can be seen from Figure 7a,b that with the increase in $\beta_{r}$ and $\beta_{d}$, the profits of both the manufacturer and the retailer under each decision scenario will increase, and no matter how $\beta_{r}$ and $\beta_{d}$ change, compared with the absence of coordination, the profits of the manufacturer and retailer under the coordination mechanism have been improved.

Figure 7c shows that when the initial reference low-carbon level is low, the profits of the manufacturer and retailer will both increase with the increase in $\alpha_{r}$. When the initial reference low-carbon level is high, the profits of the manufacturer and retailer will decrease as $\alpha_{r}$ increases. Therefore, when the initial reference low-carbon level is low, the reference low-carbon effect of the offline channel is beneficial to both parties. At this time, the manufacturer should also increase emission reduction investment. Conversely, the manufacturer should reduce emissions reduction investment as much as possible within the allowable range, but the retailer should increase investment in low-carbon publicity to reduce the negative impact on product market demand due to the reduction in emissions reduction investment of the manufacturer.

Figure 7d shows that when the initial reference low-carbon level is low, the profits of the manufacturer and retailer will both increase with the increase in $\alpha_{d}$. While when the initial reference low-carbon level is high, the profits of the manufacturer and retailer will decrease as $\alpha_{d}$ increases. Therefore, when the initial reference low-carbon level is low, the reference low-carbon effect of the online channel is beneficial to both parties. At this time, the manufacturer should also increase emission reduction investment.

The above conclusions show that in real life, when consumers’ initial reference low-carbon levels are different, manufacturers and retailers should flexibly decide their own emission reduction investment strategies. When the initial reference low-carbon level is low, manufacturers should increase emission reduction investment. When the initial reference low-carbon level is high, the manufacturer should reduce emissions reduction investment within the allowable range, but the retailer should increase investment in low-carbon publicity.

**Figure 8:**
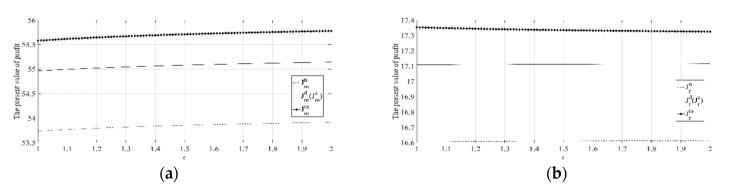
The impact of memory parameter on profits. (**a**) The effect of ε on the manufacturer’s profit. (**b**) The effect of ε on the retailer’s profit.

Figure 8a,b shows that with the increase in memory parameter ε, the manufacturer’s profit under each decision scenario will increase, the retailer’s profit under other decision scenarios will also increase, while the retailer’s profit under the coordination mechanism scenario will decrease. The above phenomenon shows that the longer the consumer’s memory of products’ low carbon levels, the higher the manufacturer’s profit will be, and the higher the retailer’s profit will be except the coordination mechanism scenario. Based on the above understanding, members of the supply chain should strengthen communication with potential consumers, stimulate potential consumers through a variety of information, strengthen their memory of enterprises’ emission reduction investment information, and realize the purpose of increasing the profits of each member by improving the memory parameter. In addition, under the coordination mechanism scenario, the manufacturer should also actively negotiate with the retailer to ensure that it can make up for the retailer’s loss of profit as much as possible, so that the coordination mechanism can proceed.

**Figure 9:**
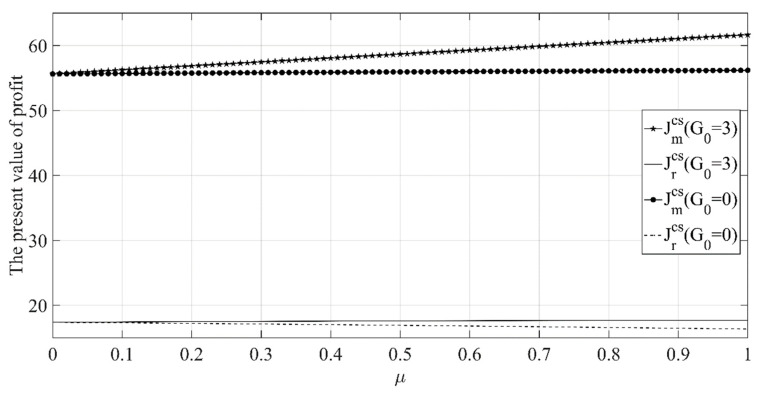
The impact of μ on profits.

Figure 9 shows that when the initial low-carbon goodwill level is high, the profits of the manufacturer and retailer will both increase with the increase in μ. While when the initial goodwill level is high, the profits of the manufacturer and retailer will decrease as μ increases. Therefore, when the initial low-carbon goodwill of the product is high, it is beneficial for both the manufacturer and the retailer to increase the consumer’s recognition of the low-carbon goodwill. At this time, the retailer should also increase investment in low-carbon promotion to maximize the impact of low-carbon goodwill on the product market demand.

## 8. Conclusions

### 8.1. Main Conclusions

Based on the market background of the low-carbon economy and the dual-channel sales model of e-commerce, this paper considers the impact of consumers’ reference low-carbon effect and product low-carbon goodwill on the low-carbon products’ sales volume of different channels and builds joint emission reduction dynamic optimization models of a dual-channel supply chain led by the manufacturer. Then, this paper uses differential game theory to solve the equilibrium strategies of the manufacturer’s emission reduction investment and the retailer’s low-carbon publicity investment, the optimal change trajectory of the product low-carbon goodwill and reference low-carbon level, the optimal profits of the manufacturer and the retailer. This paper compares and analyzes the relevant equilibrium strategies under different decision scenarios by theoretical derivation and numerical simulation and then designs a cost-sharing contract to achieve supply chain coordination. The main conclusions of this paper are as follows: 

The manufacturer’s optimal emission reduction investment under decentralized decision scenarios has a positive relationship with the two channels’ marginal profit and discount rate and has a negative relationship with the memory parameter. The retailer’s low-carbon publicity investment has a positive relationship with its marginal profit and has a negative relationship with the discount rate. Under centralized decision scenario, the manufacturer’s optimal emission reduction investment not only has a positive relationship with the marginal profit of its two channels, but also has a positive relationship with the retailer’s marginal profit. Retailers’ low-carbon publicity investment is not only positively related to its own marginal profit, but also positively related to the two-channels’ marginal profit of the manufacturer.

Under centralized decision scenario, the equilibrium strategies of the manufacturer and retailer and the overall profit of the supply chain are better than those under decentralized decision scenarios. Compared with the decentralized decision scenario without cost sharing, the added value of the retailer’s low-carbon publicity investment under the centralized decision scenario will increase with the increase in the two-channels’ marginal profit of the manufacturer, and the added value of the manufacturer’s emission reduction investment will increase with the increase in the retailer’s marginal profit. 

Under the decentralized decision scenario with cost sharing, when two-channels’ marginal profit of the manufacturer and the marginal profit of the retailer satisfy a certain relationship, the manufacturer will be willing to share a certain proportion of the low-carbon publicity investment cost for the retailer, and the cost-sharing ratio will increase with the increase in two-channels’ marginal profit of the manufacturer, while it will decline with the increase in the retailer’s marginal profit. Compared with the decentralized decision scenario without cost sharing, the cost-sharing contract can effectively promote the retailer to increase investment in low-carbon publicity, but it cannot promote the manufacturer to increase investment in emission reduction. What’s more, the profits of the manufacturer and retailer and the overall profit of the supply chain under the cost-sharing contract will be significantly improved. 

Under the decentralized decision scenario with bilateral cost sharing, when the two-channels’ marginal profit of the manufacturer and the marginal profit of the retailer satisfy a certain relationship, the retailer is also willing to share a certain proportion of the emission reduction cost for the manufacturer, and the sharing ratio will increase with the increase in the retail’s marginal profit, while it will decline with the increase in the two-channels’ marginal profit of the manufacturer. Compared with the decentralized decision scenario without cost sharing, the bilateral cost-sharing contract can effectively promote the retailer to increase investment in low-carbon publicity, the manufacturer to increase emissions reduction investment, and the profits of the manufacturer and retailer and the overall profit of the supply chain will be significantly improved.

When the low-carbon publicity cost-sharing ratio provided by the manufacturer to the retailer meets a certain relationship, and both parties adopt the equilibrium strategies under the centralized decision scenario, then the supply chain system can reach a state of coordination at this time, and both parties can achieve Pareto improvement. On the contrary, if the cost-sharing ratio cannot be within a reasonable range, the manufacturer and the retailer will not accept the contract, and the supply chain system will not be able to achieve coordination.

Different initial low-carbon goodwill and reference low-carbon level will only affect the trend of low-carbon goodwill and reference low-carbon level over time. As time goes by, low-carbon goodwill and reference low-carbon level will both reach a stable level, and the stable level is, respectively, related to the retailer’s equilibrium low-carbon publicity investment and the manufacturer’s equilibrium emission reduction investment but has nothing to do with the initial low-carbon goodwill and the reference low-carbon level.

When the initial reference low-carbon level is low, the reference low-carbon effect of the offline channel is beneficial to both parties. At this time, the manufacturer should also increase emission reduction investment. Conversely, the manufacturer should reduce emissions reduction investment as much as possible within the allowable range, but the retailer should increase investment in low-carbon publicity to reduce the negative impact on product market demand due to the reduction in emissions reduction investment of the manufacturer. When the initial low-carbon goodwill of the product is high, it is beneficial for both the manufacturer and the retailer to increase the consumer’s recognition of the low-carbon goodwill. At this time, the retailer should also increase investment in low-carbon promotion to maximize the impact of low-carbon goodwill on the product market demand. The manufacturer should also share the low-carbon promotion cost for the retailer, so as to encourage retailers to further increase investment in low-carbon promotion. When the initial low-carbon goodwill is low, consumers’ recognition of low-carbon goodwill is detrimental to both parties. At this time, manufacturers should actively bear the cost of low-carbon promotion for retailers to reduce the negative impact on the product demand due to the reduced investment in low-carbon publicity by retailers, so that it can avoid causing damage to the profits of both parties and affecting the cooperation between the two parties.

### 8.2. Management Insights

Based on the research conclusions, in order to provide more references for real-life supply chain companies to conduct joint emission reductions, we give the following management insights: 

The vertical integration of the supply chain can effectively reduce the double marginal effect generated by decentralized decision making, promote manufacturers to increase investment in emission reduction and retailers to increase investment in low-carbon publicity, improve the profit level of both parties, and help enterprises establish a competitive advantage in the increasingly fierce competition environment. At present, with the increasingly prosperous of low-carbon economy, the concept of low-carbon consumption has become increasingly popular, and the competition for low-carbon products in the market has become increasingly fierce. Meanwhile, market competition has gradually shifted from competition between individual companies to competition between supply chains. Therefore, supply chain member companies should actively engage in vertical integration, strengthen communication and collaboration, and achieve long-term and sustainable development. 

When the supply chain cannot achieve complete vertical integration, manufacturers and retailers should also actively cooperate by sharing abatement costs with each other to increase the profits of both parties. At the same time, since the cost-sharing ratio is proportional to their own marginal profit, in order to further enhance the level of cooperation between the two parties, manufacturers can improve their own marginal profits by adopting low-carbon technology upgrades and optimizing low-carbon products’ production processes. At the same time, retailers can also increase their own marginal profits by improving low-carbon advertising efficiency.

With the continuous improvement of consumers’ environmental awareness and consumption levels, they will pay more and more attention to the low-carbon quality and low-carbon goodwill of products when purchasing products. Therefore, in order to further establish a competitive advantage and achieve sustainable development, manufacturers should actively reduce carbon emissions through a variety of ways, and retailers should also actively promote low carbon through multiple channels. 

### 8.3. Research Limitations

It cannot be denied that there are still some limitations in this paper, and further research and discussion are needed. For example, this paper only considers the manufacturer’s dual-channel sales structure, but in real life, more and more retailers have also established online sales channels. In addition, there are also situations where manufacturers and retailers simultaneously open online sales channels; therefore, future research can explore the cooperative emission reduction problem of supply chain members in a more complex sales structure. In addition, this paper does not consider competition and cooperation between sales channels; however, as manufacturers or retailers open other sales channels, it is inevitable that there will be competition and cooperation between channels, therefore the competition and cooperation between sales channels can be further discussed in the future.

## Figures and Tables

**Table 1:** Some literature most relevant to this paper.

Author	Reference Low-Carbon Effect	Dual Channel	Low-Carbon Goodwill	Supply Chain Coordination
Lou et al. [40]	✓			
Zhang et al. [41]	✓		✓	
Ye et al. [42]	✓		✓	✓
Wang et al. [43]	✓			
Zhou et al. [44]		✓		✓
This paper	✓	✓	✓	✓

**Table 2:** Decision variables and major parameters.

**Decision Variables**	**The Meaning of the Decision Variables**
E(t)	Manufacturer’s emission reduction investment at time t
A(t)	Retailer’s low-carbon promotion investment at time t
**Parameters**	**The Meaning of the Parameters**
$\alpha_{d}$	Influence coefficient of reference low-carbon effect on the online demand
$\alpha_{r}$	Influence coefficient of reference low-carbon effect on the offline demand
$\beta_{r}$	Influence coefficient of manufacturer’s emission reduction on the offline demand
$\beta_{d}$	Influence coefficient of manufacturer’s emission reduction on the online demand
k	Influence coefficient of retailer’s low-carbon promotion on the offline demand
ξ	Spillover effect coefficient of retailer’s offline low-carbon promotion
ρ	Discount rate
μ	Influence coefficient of low-carbon goodwill on the demand of online and offline
$\pi_{md}$	Manufacturer’s marginal profit in online direct sales channel
$\pi_{mr}$	Manufacturer’s marginal profit in offline channel
$\pi_{rr}$	Retailer’s marginal profit in offline channel
G(t)	Low-carbon goodwill level at time t
R(t)	Reference low-carbon level at time t
$J_{m}$	Present value of the manufacturer’s profit
$J_{r}$	Present value of the retailer’s profit
$J_{sc}$	Present value of the total profit of supply chain
$\phi_{d}$	Cost-sharing rate under cost-sharing decentralized decision scenario
$\phi_{c}$, $\gamma_{c}$	Cost-sharing rates under the bilateral cost-sharing decentralized decision scenario
$\phi_{cs}$	Cost-sharing rate under the supply chain coordination mechanism
$\eta_{m}$, $\eta_{r}$	Emission reduction cost coefficient and low-carbon promotion cost coefficient.

**Table 3:** Comparative static analysis of parameters under decentralized decision scenarios.

	E	A	G	R
$\pi_{mr}$	+	×	×	+
$\pi_{md}$	+	×	×	+
$\pi_{rr}$	×	+	+	×
$\alpha_{r}$	+	×	×	+
$\alpha_{d}$	+	×	×	+
$\beta_{r}$	+	×	×	+
$\beta_{d}$	+	×	×	+
k	×	+	+	×
μ	×	+	+	×
ξ	×	×	×	×
ρ	+	−	−	+
ε	−	×	×	−
σ	×	−	−	×
$\eta_{m}$	−	×	×	−
$\eta_{r}$	×	−	−	×
λ	×	+	+	×

Note: “+” means positive correlation; “-” means negative correlation; “×” means irrelevant.

**Table 4:** Comparative static analysis of parameters under the centralized decision scenario.

	E	A	G	R
$\pi_{mr}$	+	+	+	+
$\pi_{md}$	+	+	+	+
$\pi_{rr}$	+	+	+	+
$\alpha_{r}$	+	×	×	+
$\alpha_{d}$	+	×	×	+
$\beta_{r}$	+	×	×	+
$\beta_{d}$	+	×	×	+
k	×	+	+	×
μ	×	+	+	×
ξ	×	+	+	×
ρ	+	−	−	+
ε	−	×	×	−
σ	×	−	−	×
$\eta_{m}$	−	×	×	−
$\eta_{r}$	×	−	−	×
λ	×	+	+	×

Note: “+” means positive correlation; “-” means negative correlation; “×” means irrelevant.

## Appendix A

**Proof of Theorem** **1.** According to the solution method of the optimal control problem from Equation (6), the optimal profit function of the retailer at time t is as follows:

$$\begin{array}{l} J_{r}^{n}\left(G,R,t\right)=\underset{A}{\max}\int_{t}^{\infty }e^{-\rho s}\left[\pi_{rr}\left[d_{r}+\left(\alpha_{r}+\beta_{r}\right)E+kA+\mu G-\alpha_{r}R\right]-\frac{\eta_{r}A^{2}}{2}\right]ds\\ =e^{-\rho t}\underset{A}{\max}\int_{t}^{\infty }e^{-\rho \left(s-t\right)}\left[\pi_{rr}\left[d_{r}+\left(\alpha_{r}+\beta_{r}\right)E+kA+\mu G-\alpha_{r}R\right]-\frac{\eta_{r}A^{2}}{2}\right]ds \end{array}$$

Let $V_{r}\left(G,R\right)=\underset{A}{\max}\int_{t}^{\infty }e^{-\rho \left(s-t\right)}\left[\pi_{rr}\left[d_{r}+\left(\alpha_{r}+\beta_{r}\right)E+kA+\mu G-\alpha_{r}R\right]-\frac{\eta_{r}A^{2}}{2}\right]ds$, then the retailer’s optimal profit function at time t can be expressed as: $J_{r}^{*}\left(G,R,t\right)=e^{-\rho t}V_{r}\left(G,R\right)$. According to optimal control theory, the available HJB equation is as follows:

$$\rho V_{r}\left(G,R\right)=\underset{A}{\max}\left[\pi_{rr}\left[d_{r}+\left(\alpha_{r}+\beta_{r}\right)E+kA+\mu G-\alpha_{r}R\right]-\frac{\eta_{r}A^{2}}{2}+\frac{\partial V_{r}}{\partial R}\left(\varepsilon \left(E-R\right)\right)+\frac{\partial V_{r}}{\partial G}\left(\lambda A-\sigma G\right)\right]$$

Solve the first-order partial derivative of A with respect to the right part of the above equation and make it equal to 0, we can get:

$$A=\frac{\pi_{rr}k+\lambda \frac{\partial V_{r}}{\partial G}}{\eta_{r}}$$

Then, solve the manufacturer’s optimal control problem. The manufacturer will determine its own emission reduction strategy based on the retailer’s optimal feedback strategy. From Equation (5), we can see that the manufacturer’s optimal profit function at time t is: $J_{m}^{*}\left(G,R,t\right)=e^{-\rho t}V_{m}\left(G,R\right)$. Similarly, let $V_{m}\left(G,R\right)=\underset{E}{\max}\int_{t}^{\infty }e^{-\rho \left(s-t\right)}\left[\pi_{mr}\left[\begin{array}{l} d_{r}+\left(\alpha_{r}+\beta_{r}\right)E\\ +\mu G-\alpha_{r}R+kA \end{array}\right]+\pi_{md}\left[\begin{array}{l} d_{d}+\left(\alpha_{d}+\beta_{d}\right)E\\ +\mu G+\xi kA-\alpha_{d}R \end{array}\right]-\frac{\eta_{m}E^{2}}{2}\right]ds$. At this time, the manufacturer’s optimal control problem satisfies the following HJB equation:

$$\rho V_{m}\left(G,R\right)=\underset{E}{\max}\left[\begin{array}{l} \pi_{mr}\left[\begin{array}{l} d_{r}+\left(\alpha_{r}+\beta_{r}\right)E+kA\\ +\mu G-\alpha_{r}R \end{array}\right]+\pi_{md}\left[\begin{array}{l} d_{d}+\left(\alpha_{d}+\beta_{d}\right)E\\ +\mu G+\xi kA-\alpha_{d}R \end{array}\right]-\frac{\eta_{m}E^{2}}{2}+\frac{\partial V_{m}}{\partial R}\left(\varepsilon \left(E-R\right)\right)\\ +\frac{\partial V_{m}}{\partial G}\left(\lambda A-\sigma G\right) \end{array}\right]$$

Solve the first-order partial derivative of E with respect to the right part of the above equation and make it equal to 0, we can get:

$$E=\frac{\pi_{mr}\left(\alpha_{r}+\beta_{r}\right)+\pi_{md}\left(\alpha_{d}+\beta_{d}\right)+\varepsilon \frac{\partial V_{m}}{\partial R}}{\eta_{m}}$$

After sorting, we can get:

$$\begin{array}{l} \rho V_{r}\left(G,R\right)=\left(\pi_{rr}\mu -\sigma \frac{\partial V_{r}}{\partial G}\right)G-\left(\pi_{rr}\alpha_{r}+\varepsilon \frac{\partial V_{r}}{\partial R}\right)R+\frac{{\left(\pi_{rr}k+\lambda \frac{\partial V_{r}}{\partial G}\right)}^{2}}{2\eta_{r}}+\pi_{rr}d_{r}\\ +\frac{\left[\pi_{rr}\left(\alpha_{r}+\beta_{r}\right)+\varepsilon \frac{\partial V_{r}}{\partial R}\right]\left[\pi_{mr}\left(\alpha_{r}+\beta_{r}\right)+\pi_{md}\left(\alpha_{d}+\beta_{d}\right)+\varepsilon \frac{\partial V_{m}}{\partial R}\right]}{\eta_{m}} \end{array}\;\begin{array}{l} \rho V_{m}\left(G,R\right)=\left(\begin{array}{l} \pi_{mr}\mu +\pi_{md}\mu \\ -\sigma \frac{\partial V_{m}}{\partial G} \end{array}\right)G-\left(\begin{array}{l} \pi_{mr}\alpha_{r}+\pi_{md}\alpha_{d}\\ +\varepsilon \frac{\partial V_{m}}{\partial R} \end{array}\right)R+\left(\pi_{mr}k+\pi_{md}\xi k+\lambda \frac{\partial V_{m}}{\partial G}\right)\frac{\left(\pi_{rr}k+\lambda \frac{\partial V_{r}}{\partial G}\right)}{\eta_{r}}\\ +\frac{{\left[\pi_{mr}\left(\alpha_{r}+\beta_{r}\right)+\pi_{md}\left(\alpha_{d}+\beta_{d}\right)+\varepsilon \frac{\partial V_{m}}{\partial R}\right]}^{2}}{2\eta_{m}}+\pi_{mr}d_{r}+\pi_{md}d_{d} \end{array}$$

Suppose the specific expressions of $V_{r}\left(G,R\right)$, $V_{m}\left(G,R\right)$ are as follows:

$$\begin{array}{l} V_{r}\left(G,R\right)=b_{1}G+c_{1}R+f_{1}\;\\ V_{m}\left(G,R\right)=b_{2}G+c_{2}R+f_{2} \end{array}$$

where $b_{1}$, $c_{1}$$f_{1}\;$, $b_{2}$, $c_{2}$, $f_{2}\;$ are unknown constants. Sort and compare coefficients of similar terms, we can get the constraint equation system including $b_{1}$, $c_{1}$, $f_{1}$, $b_{2}$, $c_{2}$, $f_{2}\;$ is as follows:

$$\left\{ \begin{array}{l} \rho b_{1}=\pi_{rr}\mu -\sigma b_{1}\\ \rho c_{1}=-\pi_{rr}\alpha_{r}-\varepsilon c_{1}\\ \rho f_{1}=\pi_{rr}d_{r}+\frac{{\left(\pi_{rr}k+\lambda b_{1}\right)}^{2}}{2\eta_{r}}+\frac{\left[\pi_{rr}\left(\alpha_{r}+\beta_{r}\right)+\varepsilon c_{1}\right]\left[\pi_{mr}\left(\alpha_{r}+\beta_{r}\right)+\pi_{md}\left(\alpha_{d}+\beta_{d}\right)+\varepsilon c_{2}\right]}{\eta_{m}}\\ \rho b_{2}=\pi_{mr}\mu +\pi_{md}\mu -\sigma b_{2}\\ \rho c_{2}=-\pi_{mr}\alpha_{r}-\pi_{md}\alpha_{d}-\varepsilon c_{2}\\ \rho f_{2}=\pi_{mr}d_{r}+\pi_{md}d_{d}+\left(\pi_{mr}k+\pi_{md}\xi k+\lambda b_{2}\right)\frac{\left(\pi_{rr}k+\lambda b_{1}\right)}{\eta_{r}}+\frac{{\left[\pi_{mr}\left(\alpha_{r}+\beta_{r}\right)+\pi_{md}\left(\alpha_{d}+\beta_{d}\right)+\varepsilon c_{2}\right]}^{2}}{2\eta_{m}} \end{array}\right.$$

Solving the above equations, we can get:

$$b_{1}^{*}=\frac{\pi_{rr}\mu }{\rho +\sigma },c_{1}^{*}=-\frac{\pi_{rr}\alpha_{r}}{\rho +\varepsilon },b_{2}^{*}=\frac{\pi_{mr}\mu +\pi_{md}\mu }{\rho +\sigma },c_{2}^{*}=\frac{-\pi_{mr}\alpha_{r}-\pi_{md}\alpha_{d}}{\rho +\varepsilon },\;f_{1}^{*}=\frac{1}{\rho }\left\{\begin{array}{l} \frac{\left[\pi_{rr}\left[\rho \left(\alpha_{r}+\beta_{r}\right)+\varepsilon \beta_{r}\right]\right]\left[\pi_{mr}\left[\rho \left(\alpha_{r}+\beta_{r}\right)+\varepsilon \beta_{r}\right]+\pi_{md}\left[\rho \left(\alpha_{d}+\beta_{d}\right)+\varepsilon \beta_{d}\right]\right]}{\eta_{m}{\left(\rho +\varepsilon \right)}^{2}}+\pi_{rr}d_{r}\\ +\frac{{\left[\left(\rho +\sigma \right)k\pi_{rr}+\lambda \pi_{rr}\mu \right]}^{2}}{2\eta_{r}{\left(\rho +\sigma \right)}^{2}} \end{array}\right\},\;f_{2}^{*}=\frac{1}{\rho }\left\{\begin{array}{l} \pi_{mr}d_{r}+\pi_{md}d_{d}+\frac{{\left[\pi_{mr}\left[\rho \left(\alpha_{r}+\beta_{r}\right)+\varepsilon \beta_{r}\right]+\pi_{md}\left[\rho \left(\alpha_{d}+\beta_{d}\right)+\varepsilon \beta_{d}\right]\right]}^{2}}{2\eta_{m}{\left(\rho +\varepsilon \right)}^{2}}\\ +\frac{\pi_{rr}\left[\left(\rho +\sigma \right)k+\lambda \mu \right]\left[k\left(\rho +\sigma \right)\left(\pi_{mr}+\xi \pi_{md}\right)+\lambda \mu \left(\pi_{mr}+\pi_{md}\right)\right]}{\eta_{r}{\left(\rho +\sigma \right)}^{2}} \end{array}\right\}.$$

We can get the specific expressions of $V_{r}\left(G,R\right)$, $V_{m}\left(G,R\right)$:

$$\begin{array}{l} V_{r}\left(G,R\right)=b_{1}^{*}G+c_{1}^{*}R+\;f_{1}^{*}\\ V_{m}\left(G,R\right)=b_{2}^{*}G+c_{2}^{*}R+f_{2}^{*} \end{array}$$

Then, we can get the equilibrium strategies of the retailer and manufacturer:

$$\left\{ \begin{array}{l} E^{n*}=\frac{\pi_{mr}\left[\rho \left(\alpha_{r}+\beta_{r}\right)+\varepsilon \beta_{r}\right]+\pi_{md}\left[\rho \left(\alpha_{d}+\beta_{d}\right)+\varepsilon \beta_{d}\right]}{\eta_{m}\left(\rho +\varepsilon \right)}\\ A^{n*}=\frac{\pi_{rr}\left[k\left(\rho +\sigma \right)+\lambda \mu \right]}{\eta_{r}\left(\rho +\sigma \right)} \end{array}\right.$$

□ So far, Theorem 1 (1) is proved, and then, according to the boundary conditions of the low-carbon goodwill $G\left(0\right)=G_{0}$, the optimal trajectory of low-carbon goodwill can be obtained, as shown in Theorem 1 (2), and then, according to the boundary conditions of the consumers’ reference low-carbon level $R\left(0\right)=R_{0}$, the optimal trajectory of the consumer’s reference low-carbon level can be obtained, as shown in Theorem 1 (3). Finally, the above results are substituted into the expressions of the optimal profit functions of the manufacturer and retailer; the optimal profit functions of the manufacturer and retailer are shown in Theorem (4). Theorem 1 is proved.
